# STAT3-mediated IGF-2 secretion in the tumour microenvironment elicits innate resistance to anti-IGF-1R antibody

**DOI:** 10.1038/ncomms9499

**Published:** 2015-10-14

**Authors:** Ji-Sun Lee, Ju-Hee Kang, Hye-Jin Boo, Su-Jung Hwang, Sungyoul Hong, Su-Chan Lee, Young-Jun Park, Tae-Moon Chung, Hyewon Youn, Seung Mi Lee, Byoung Jae Kim, June-Key Chung, Yeonseok Chung, William N. William, Young Kee Shin, Hyo-Jong Lee, Seung-Hyun Oh, Ho-Young Lee

**Affiliations:** 1 College of Pharmacy and Research Institute of Pharmaceutical Sciences, Seoul National University, 1 Gwanak-ro, Gwanak-gu, Seoul 151 742, Korea; 2 National Cancer Center, Goyang-si, Gyeonggi-do 410 769, Korea; 3 College of Pharmacy, Inje University, Gimhae, Gyeongnam 621 749, Korea; 4 Department of Nuclear Medicine, Cancer Imaging Center, Seoul National University Hospital, Seoul 110 744, Korea; 5 Department of Obstetrics and Gynecology, Seoul Metropolitan Government Seoul National University Boramae Medical Center, Seoul 156 707, Korea; 6 Department of Obstetrics and Gynecology, Seoul National University College of Medicine, Seoul 110 744, Korea; 7 Department of Thoracic/Head and Neck Medical Oncology, The University of Texas M.D. Anderson Cancer Center, Houston, Texas 77030, USA; 8 The Center for Anti-Cancer CDx, N-Bio, Seoul National University, Seoul 151 742, Korea; 9 College of Pharmacy, Gachon University, Inchon 406 840, Korea

## Abstract

Drug resistance is a major impediment in medical oncology. Recent studies have emphasized the importance of the tumour microenvironment (TME) to innate resistance, to molecularly targeted therapies. In this study, we investigate the role of TME in resistance to cixutumumab, an anti-IGF-1R monoclonal antibody that has shown limited clinical efficacy. We show that treatment with cixutumumab accelerates tumour infiltration of stromal cells and metastatic tumour growth, and decreases overall survival of mice. Cixutumumab treatment stimulates STAT3-dependent transcriptional upregulation of IGF-2 in cancer cells and recruitment of macrophages and fibroblasts via paracrine IGF-2/IGF-2R activation, resulting in the stroma-derived CXCL8 production, and thus angiogenic and metastatic environment. Silencing IGF-2 or STAT3 expression in cancer cells or IGF-2R or CXCL8 expression in stromal cells significantly inhibits the cancer–stroma communication and vascular endothelial cells’ angiogenic activities. These findings suggest that blocking the STAT3/IGF-2/IGF-2R intercellular signalling loop may overcome the adverse consequences of anti-IGF-1R monoclonal antibody-based therapies.

## Abstract (web summary)

Cixutumumab is an anti-IGF-1R monoclonal antibody and is used to treat cancer; however, tumours can develop resistance to the therapy. Here, the authors show that the resistance is mediated by activation of STAT3 that results in an IGF2/IGF-2R signalling loop and recruitment of macrophages.

## Graphical abstract

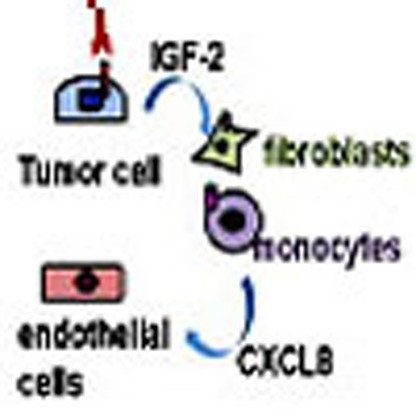

Despite the enormous efforts to incorporate new drugs into clinical practice and large repertoire of anticancer therapies available, a major challenge to cancer treatment is drug resistance. Hence, the rational design of anticancer therapies should include strategies that circumvent treatment-associated drug resistance. The insulin-like growth factor (IGF) axis is regulated by a complex interplay between ligands, cognate receptors and binding proteins. This axis has been proposed as one of the most promising targets for anticancer therapies. A number of clinical trials with IGF-1 receptor (IGF-1R)-targeted therapies, mostly using monoclonal antibodies, have sought to abrogate IGF-1R function in various cancers^1^,^2^. However, the overall response rate to the therapy has been underwhelming and enthusiasm for the therapy has waned^3^,^4^,^5^,^6^. Accordingly, efforts have focused on understanding mechanisms underlying resistance against anti-IGF-1R monoclonal antibody-based therapies.

Several preclinical studies have proposed mechanisms underlying emergent resistance to the anti-IGF-1R therapies^7^,^8^,^9^,^10^. We have demonstrated a critical role for integrin and epidermal growth factor receptor (EGFR) signalling in inherent resistance of cancer cells to cixutumumab, a fully human IgG1 monoclonal antibody against IGF-1R^11^. These studies may explain the mechanisms underlying cancer cells’ resistance to anti-IGF-1R. However, solid tumours exhibit an organ-like structure, consisting of various cell types including cancer cells, tumour-associated fibroblasts, infiltrating immune cells and endothelial cells^12^. Hence, such unicellular mechanisms may explain only part of the events underlying resistance to anti-IGF-1R monoclonal antibodies. Indeed, considerable debate surrounds the role of the tumour microenvironment (TME) in tumour response to therapies^13^. Recent studies have implicated adhesion molecules and growth factors secreted by tumour or stromal cells through autocrine, paracrine or endocrine production in anticancer drug resistance^14^,^15^. In addition, the growth- , angiogenesis- and metastasis-promoting impacts of the TME have been noted^16^,^17^,^18^.

In this study, we performed a series of experiments to elucidate the possible role of the TME in responsiveness to anti-IGF-1R therapies. Here we report that pharmacological or genomic blockade of IGF-1R induces a protective reprogramming of cancer cells to stimulate signal transducer and activator of transcription 3 (STAT3)-dependent transcriptional increases in IGF-2 in cancer cells, promoting tumour–stromal communication through IGF-2R-dependent paracrine signalling. The resultant stromal production of several cytokines, especially CXCL8, provides proangiogenic signals and increases metastatic potential in tumours. Our data suggest that the dual inhibition of IGF-1R and either STAT3 or IGF-2 may serve as a therapeutic strategy to overcome resistance to anti-IGF-1R monoclonal antibody-based therapies.

## Results

### Increased cancer invasiveness after ablation of IGF-1R

Several clinical trials have evaluated the therapeutic activities of IGF-1R monoclonal antibody in various types of cancers including breast cancer, non-small cell lung cancer (NSCLC) and head and neck squamous cell carcinoma (HNSCC)^19^,^20^,^21^. To assess the response of various cancer cells to an IGF-1R blockade, we evaluated the effects of cixutumumab on immune-deficient mice harbouring orthotopic tumours of luciferase (Luc)-expressing MDA231D3H2LN (MDA231), H1299 or 686LN cells, as three representative human cell lines for breast cancer, NSCLC and HNSCC, respectively.

**Figure 1:**
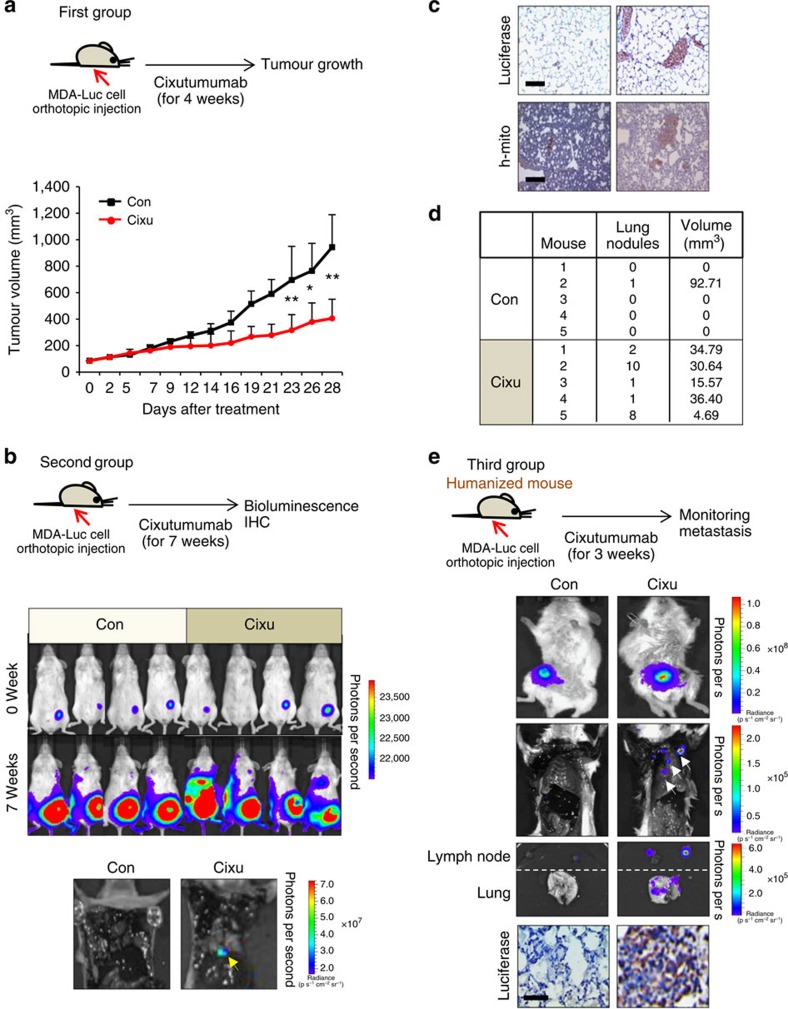
Increased metastasis after cixutumumab treatment in orthotopic breast tumour models. (**a**) Breast cancer cells (MDA231–Luc) were orthotopically injected into Balb/c nude mice (*n*=9 per group) and the mice were treated with cixutumumab (10 mg kg^−1^, intraperitoneally, once weekly). Tumour volumes were measured over 4 weeks of drug treatment. Data are presented as the mean tumour volume±s.d. (**b**) MDA231–Luc cells were orthotopically injected into NOD/SCID mice (*n*=5 per group) and the mice were treated with cixutumumab (10 mg kg^−1^, intraperitoneally, once weekly) for 7 weeks. Top: representative bioluminescence images visualizing the tumour cells on week 0 and week 7. Bottom: bioluminescence images of a representative mouse in each group after killing. (**c**) Representative images of excised lungs are presented to confirm the presence of metastasis by anti-luciferase and anti-human mitochondria protein immunostaining (Scale bar, 200 μm). (**d**) The number and volume of metastatic lung nodules were scored. (**e**) MDA231–Luc cells were orthotopically injected into NOD/SCID/JAK3^null^ mice with acquired human immune system (*n*=2 per group) and the mice were treated with cixutumumab (10 mg kg^−1^, intraperitoneally, once weekly) for 3 weeks. Representative images of live mouse (top), killed mouse, excised tissues (middle) and anti-luciferase immunostaining images of excised lung (bottom). Scale bar, 50 μm; × 400 magnification. White arrows indicate luciferase signal of metastatic tumours. **P<0.01 and ***P<0.001 by two-sided Student’s *t*-test. Cixu, cixutumumab; Con, control.

Over the 4 weeks of cixutumumab treatment, nude mice bearing MDA231–Luc tumours in the first group exhibited a significantly reduced level of tumour growth when compared with vehicle-treated control mice (Fig. 1a). Postmortem analyses of these mice also revealed no detectable metastatic tumour nodules. We then assessed the persistence of the antitumour activities of the cixutumumab treatment in the second group of non-obese diabetic (NOD)/severe combined immune-deficient (SCID) mice carrying MDA231–Luc orthotopic tumours. Surprisingly, bioluminescence imaging analysis after 7 weeks of the cixutumumab treatment provided results that suggested metastatic tumours (Fig. 1b, top). A representative cixutumumab-treated mouse, wherein the primary tumours were surgically removed, revealed a clear bioluminescence signal in the lung (Fig. 1b, bottom). We confirmed lung metastases in the cixutumumab-treated mice by means of immunohistochemical (IHC) staining of the lungs using anti-luciferase and anti-human mitochondria protein antibodies (Fig. 1c). Microscopic analyses revealed a 100% lung tumour incidence with greater levels of multiplicity and volume in the cixutumumab-treated mice than in the control mice (Fig. 1d). No detectable metastatic tumour nodules were observed in other organs.

As the immune-compromised mice may not show the same results as in immune-competent hosts, we also assessed the effects of cixutumumab on orthotopic MDA231–Luc tumours in NOD/SCID/JAK3^null^ mice, in which the human-acquired lymphoid system is reconstituted. In this model, human CD34+ haematopoietic stem cells purified from human cord blood were transplanted into the mice as described previously^22^. We confirmed the presence of human CD45+ human leukocytes in the peripheral blood collected at 4 weeks after transplantation and in the spleen collected at the time of killing (Supplementary Fig. 1). As with the findings in the NOD/SCID mouse model, bioluminescence imaging and IHC analyses revealed lung and lymph metastasis in the humanized mice after cixutumumab treatment (Fig. 1e).

**Figure 2:**
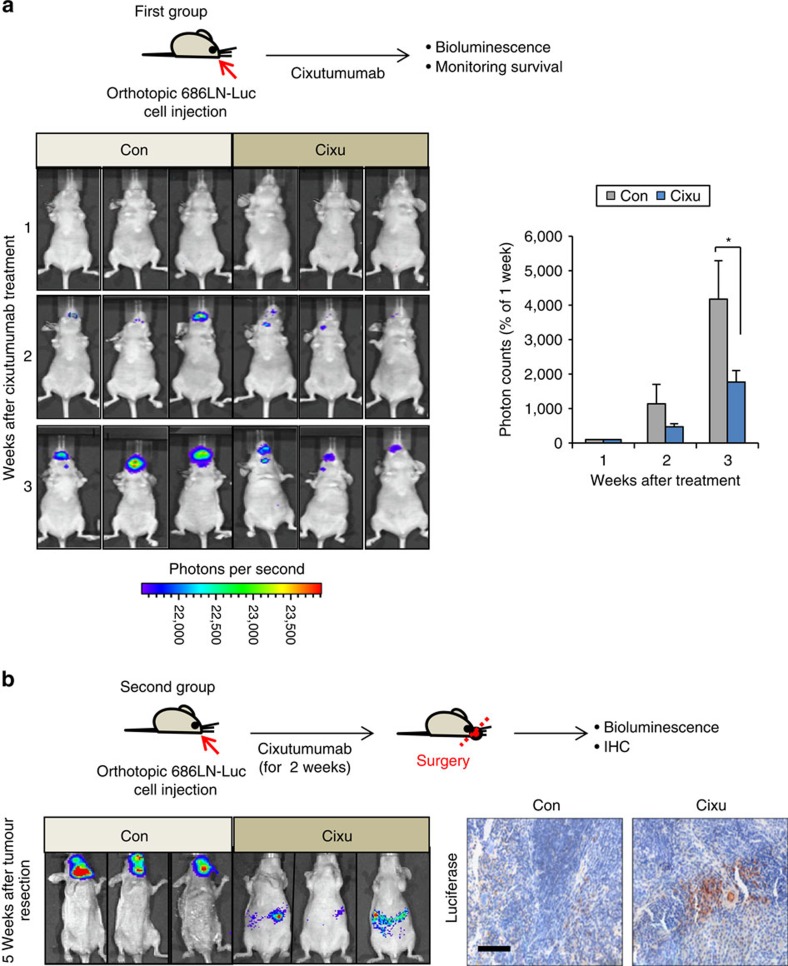
Increased metastasis after cixutumumab treatment in orthotopic HNSCC tumour models. (**a**) 686LN–Luc cells were orthotopically injected into Balb/c nude mice (*n*=8 per group) and the mice were treated with cixutumumab (10 mg kg^−1^, intraperitoneally, once weekly). Left: representative bioluminescence images visualizing the tumours. Right: quantification of bioluminescence. Data are presented as mean relative unit of photons per second ±s.d. (**b**) 686LN–Luc cells were orthotopically injected into Balb/c nude mice (*n*=10 per group). Two weeks after cixutumumab treatment (10 mg kg^−1^, intraperitoneally, once weekly), the primary tumours were surgically removed. Left: representative bioluminescence images visualizing the tumours are shown. Right: representative images of excised spleen are shown to confirm the presence of metastatic tumours using anti-luciferase immunostaining (scale bar, 200 μm). Cixu, cixutumumab; Con, control.

When mice bearing H1299–Luc tumours were analysed, a remarkably reduced survival rate was noted in the cixutumumab-treated mice compared with vehicle-treated control mice (Supplementary Fig. 2a). Bioluminescence images clearly showed a primary lung tumour, although metastatic tumours could not be seen in other organs. We measured *ex vivo* bioluminescence imaging of each organ and observed spleen metastasis (Supplementary Fig. 2b). We confirmed this finding of spleen metastasis by using IHC on four surviving mice that were receiving cixutumumab (Supplementary Fig. 2b). When nude mice bearing 686LN–Luc orthotopic tumours were analysed, the cixutumumab-treated mice also exhibited a notably decreased survival rate when compared with the control mice (Supplementary Fig. 3). In contrast, other mice bearing the same tumours in a different group exhibited a significantly reduced rate of tumour growth after cixutumumab treatment (Fig. 2a). The metastatic phenotypes of the mice in the second group were then assessed, with the primary 686LN–Luc orthotopic tongue tumours being surgically removed after 2 weeks of cixutumumab treatment. Bioluminescence (Fig. 2b, left) and IHC (Fig. 2b, right) analyses revealed spleen metastasis in these mice at 5 weeks after the tumour resection. None of the control mice displayed metastases in any organs. Therefore, it could be established that the IGF-1R blockade appeared to increase cancer metastasis in animal models of three different types of human cancers.

### The IGF-1R blockade alters the TME

We investigated the mechanisms underlying the IGF-1R blockade-induced cancer metastasis. We first determined whether the IGF-1R blockade-induced cancer metastasis was mediated by the direct effects of cixutumumab on cancer cells. However, we observed that 25 μg ml^−1^ cixutumumab, which almost completely abolished the IGF-1R expression, did not affect the proliferation, migration and expression of genes involved in the metastasis (*MMP-2* and *MMP-9*) of MDA231, H1299 and 686LN cells (Supplementary Fig. 4). The epithelial–mesenchymal transition contributes to cancer cell metastasis and the emergence of drug resistance^23^,^24^,^25^,^26^,^27^. However, no consistent changes in various epithelial–mesenchymal transition and stemness markers (E-cadherin, N-cadherin, vimentin, snail, slug, transforming growth factor-β, ABCC1, ABCG2 and ESA) were detected in H1299 and MDA231 cells during 24 days of cixutumumab treatment (Supplementary Fig. 5). Therefore, we could determine that the observed cancer metastasis was not likely to be due to the direct effects of cixutumumab on cancer cells.

**Figure 3:**
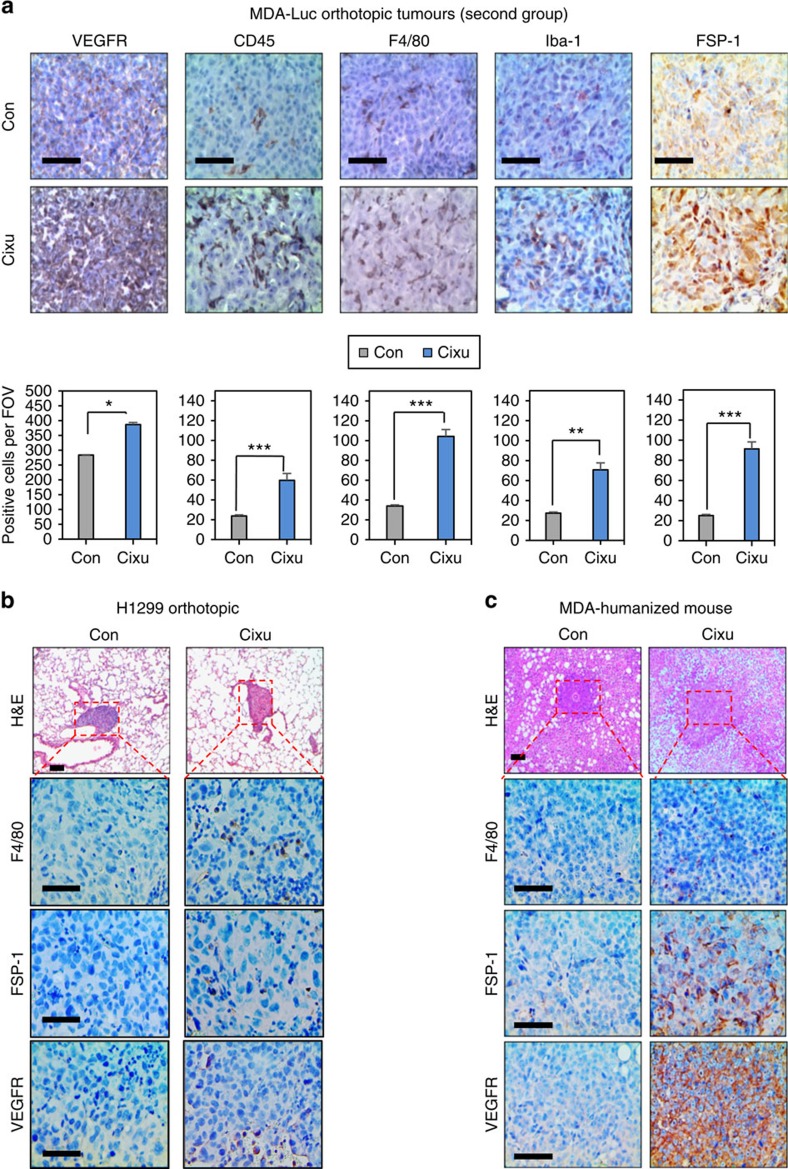
Effects of IGF-1R blockade on stromal cell recruitment *in vivo*. (**a**) Excised primary injected tumours from MDA231–Luc orthotopic tumour models were immunostained with several antibodies to verify the recruitment of endothelial cells (anti-VEGFR), leukocytes (anti-CD45), macrophages (anti-F4/80, anti-Iba-1) and fibroblasts (anti-FSP-1) in mammary fat pad. Each bar on the graph below indicates the number of positive cells per field of view (FOV). *P<0.05, **P<0.01 and ***P<0.001 by two-sided Student’s *t*-test. (**b**) Representative haematoxylin and eosin (H&E) staining and immunostaining images of excised primary lung tumours from H1299 orthotopic tumour models. (**c**) Representative H&E staining and immunostaining images of excised primary injected tumours from humanized mouse model. Scale bar, 100 μm for H&E staining images, 50 μm for immnunostaining images. Cixu, cixutumumab; Con, control.

As cixutumumab fail to change cancer cells into a more aggressive phenotype, we then hypothesized that cixutumumab may alter the TME to establish cancer-promoting conditions. As communication between cancer and stromal cells has been implicated in cancer progression^28^,^29^, we searched for the possibility of tumour infiltration of stromal cells into the collected tumours (Fig. 1 and Supplementary Fig. 2). Indeed, the MDA231–Luc tumours from cixutumumab-treated mice exhibited significantly increased levels of VEGFR^+^ vascular endothelial (VE) cells, CD45^+^ leukocytes, F4/80^+^ or Iba-1^+^ macrophages and FSP-1^+^ cancer-associated fibroblasts (Fig. 3a). These results of increased macrophage, fibroblast and VE cell infiltrations were also observed in the H1299–Luc tumours in nude mice (Fig. 3b) and the MDA231–Luc tumours in the humanized mice (Fig. 3c). Therefore, it could be seen that the IGF-1R blockade appeared to induce the tumour infiltration of various stromal cells.

### IGF-1R blockade indirectly stimulates tumour angiogenesis

**Figure 4:**
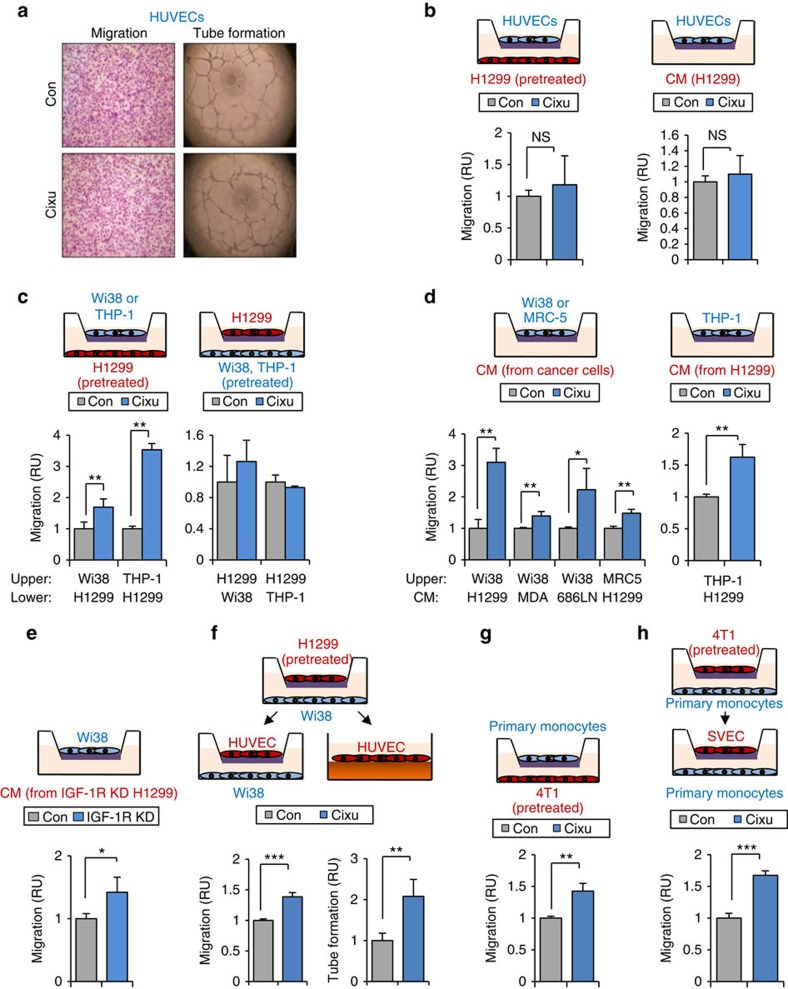
Interaction between cixutumumab-treated cancer cells and stromal cells stimulates tumour angiogenesis. (**a**) Representative images of migration and tube formation assay of cixutumumab-treated HUVECs. (**b**) HUVECs were seeded in the top chamber of the transwell insert and allowed to migrate for 6 h. Left: cixutumumab-treated H1299 cells were seeded in the bottom chambers of the transwell. Right: CM from cixutumumab-treated H1299 cells were filled in the bottom chambers of the transwell. (**c**) Co-culture of cancer cells and stromal cells. Left: Wi38 or THP-1 cells were seeded in the top chamber of the transwell insert. Cixutumumab-treated H1299 cells were seeded in the bottom chambers of the transwell. Indicated stromal cells were allowed to migrate for 16–206 h. Right: H1299 cells were seeded in the top chamber of the transwell insert. Cixutumumab-treated Wi38 or THP-1 cells were seeded in the bottom chambers of the transwell. H1299 cells were allowed to migrate for 16 h. (**d**, left) Wi38 and MRC-5 cells were seeded in the top chamber of the transwell insert. The bottom chambers were filled with CM from cixutumumab-treated cancer cells. Wi38 or MRC-5 cells were allowed to migrate for 16–20 h. (**d**, right) THP-1 cells were seeded in the top chamber of the transwell insert. The bottom chambers were filled with CM from cixutumumab-treated H1299 cells. THP-1 cells were allowed to migrate for 20 h. (**e**) The bottom chambers were filled with CM from H1299 cells with reduced IGF-1R expression. Wi38 cells were seeded in the top chamber of the transwell insert and allowed to migrate for 16 h. (**f**) Cixutumumab-treated H1299 cells were cocultured with Wi38 cells in the transwell for 24 h. Left: HUVEC cells were seeded in the new top chamber and allowed to migrate for 6 h. Right: HUVEC cells were seeded onto a Matrigel-coated 96-well plate and incubated with CM from Wi38 cells for 10 h. (**g**) Cixutumumab-treated 4T1–Luc cells were seeded in the bottom chambers of the transwell and primary monocytes were incubated in the top chamber of the transwell for 24 h. (**h**) Primary monocytes were co-cultured with cixutumumab-treated 4T1–Luc cells for 24 h. SVECs were seeded in the new top chamber and allowed to migrate for 6 h. Each bar represents the mean relative unit (RU) ±s.d. of three or four identical wells of a single representative experiment. *P<0.05, **P<0.01 and ***P<0.001 by two-sided Student’s *t*-test. NS, non significant; Cixu, cixutumumab (25 μg ml^−1^, 6 days for cell treatment); Con, control.

We also investigated whether infiltrating stromal cells contribute to cancer metastasis within an IGF-1R blockade. Based on the increased numbers of VE cells in the cixutumumab-treated tumours (Fig. 3) and the essential role of angiogenesis in the metastatic pathway^30^, we hypothesized that tumour angiogenesis could have contributed to the observed level of metastasis during the IGF-1R blockade. We then assessed the direct effects of cixutumumab on VE cells. However, the migration, tube formation, proliferation and expression of various genes involved in the angiogenesis (*VEGF*, *VEGFR-1*, *bFGF*, *PDGF-A* and *PDGF-B*) of human umbilical VE cells (HUVECs) remained unchanged after the drug treatment (Fig. 4a and Supplementary Fig. 6). Our next step was to determine whether angiogenesis is stimulated by tumour-secreted pro-angiogenic factors^31^,^32^. However, neither a co-culture with cixutumumab-treated H1299 cells nor an incubation with conditioned medium (CM) from the H1299 cells significantly changed HUVEC migration (Fig. 4b). It was consistently shown that the gene expression of the aforementioned angiogenic factors was not significantly increased in the cixutumumab-treated cancer cells (Supplementary Fig. 7). Therefore, neither autocrine production of VE cells nor direct communication between VE and cancer cells appeared to cause the tumour angiogenesis.

Analysis was then carried out on the effects of an IGF-1R blockade on stromal cells. We found that the proliferation and migration of fibroblast (Wi38) and monocyte (THP-1) cell lines were not significantly changed by cixutumumab treatment (Supplementary Fig. 8). However, a co-culturing system revealed significantly increased Wi38 and THP-1 cell migration towards the cixutumumab-pretreated H1299 cells, whereas the cixutumumab-pretreated stromal cells had a minimal impact on H1299 cell migration (Fig. 4c). CM from the cixutumumab-treated H1299, MDA231 and 686LN cells also increased the stromal cell migration compared with CM from untreated cells (Fig. 4d). Similar results were obtained by using CM from H1299 cells, in which the IGF-1R expression was impaired by short hairpin RNA (shRNA) (Fig. 4e), indicating that this event is not limited to pharmacological ablation of IGF-1R. This led us to think that the stromal cells recruited in the TME potentially could have influenced VE cells. Indeed, Wi38 cells exposed to cixutumumab-treated cancer cells significantly increased HUVEC migration and tube formation when compared with vehicle-treated control cells (Fig. 4f). We further confirmed that migration of primary monocytes isolated from nude mice bearing 686LN tumours significantly increased towards cixutumumab-pretreated 4T1 mouse breast cancer cells (Fig. 4g and Supplementary Fig. 9). In addition, primary monocytes exposed to cixutumumab-treated cancer cells significantly increased the migration of murine SV40 transformed endothelial cells (SVEC) (Fig. 4h). We confirmed the ability of cixutumumab to inhibit murine IGF-1R expression in 4T1–Luc cells and SVEC (Supplementary Fig. 10). Together, these findings suggest that IGF-1R blockade can promote communication between cancer and stromal cells, thereby promoting tumour angiogenesis.

### Cancer and stroma interact through the IGF-2/IGF-2R pathway

**Figure 5:**
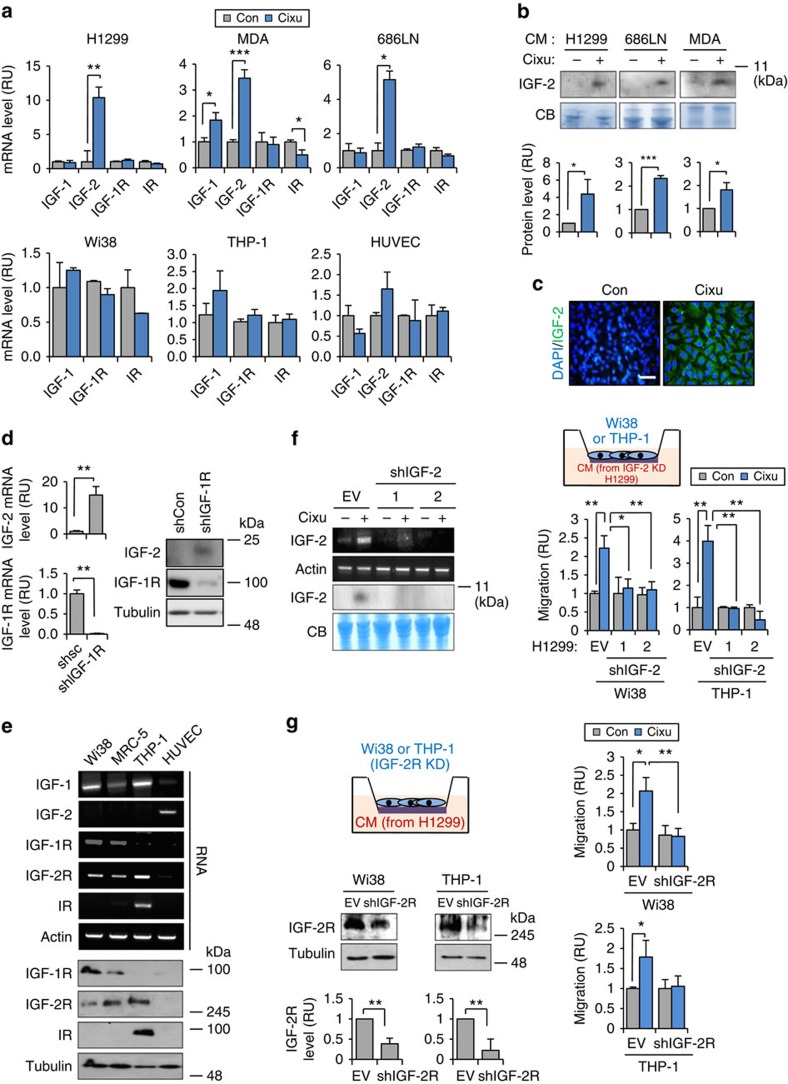
Transcriptionally upregulated IGF-2 mediates the interaction between cancer cells and stromal cells during IGF-1R blockade. (**a**) Indicated cancer cells and stromal cells were treated with cixutumumab for 6 days, and expression of ligands and receptors of the IGF axis were determined by real-time PCR. (**b**) CM from cixutumumab-treated cancer cells were analysed by western blotting to confirm IGF-2 secretion during cixutumumab treatment. Lower blots present Coomassie Blue (CB) staining as a loading control. Graph below shows densitometric analysis of three independent western blot assays. (**c**) IGF-2 immunofluorescence staining in cixutumumab-treated MDA231 cells (scale bar, 10 μm). (**d**) IGF-1R expression was stably reduced by shRNA in H1299 cells, and the expression of IGF-2 and IGF-1R was determined by real-time PCR (left) and western blotting (right). (**e**) Ligand, receptor and IGF-2R expression in stromal cells were determined by RT–PCR and western blotting. (**f**, left) RT–PCR and western blotting data revealing altered cixutumumab-induced IGF-2 expression. (**f**, right) Wi38 or THP-1 cells were seeded in the top chamber of the transwell insert. The bottom chambers were filled with CM from cixutumumab-treated H1299 cells transfected with empty vector or shIGF-2. Wi38 or THP-1 cells were allowed to migrate for 16–20 h. (**g**, left) IGF-2R expression was stably reduced by shRNA in Wi38 and THP-1 cells as demonstrated by western blotting and densitometric analysis of three independent experiment. (**g**, right) Transfected Wi38 or THP-1 cells were seeded in the top chamber of the transwell insert. The bottom chambers were filled with CM from cixutumumab-treated H1299 cells. Wi38 or THP-1 cells were allowed to migrate for 16–20 h. Each bar represents the mean relative unit (RU) ±s.d. of three replicates of a single representative experiment. *P<0.05, **P<0.01 and ***P<0.001 by two-sided Student’s *t*-test. Cixu, cixutumumab (25 μg ml^−1^, 6 days for cell treatment); Con, control; EV, empty vector.

The factors involved in cixutumumab-induced stromal cell recruitment were then determined. Based on the previous findings including: (1) functional involvement of the insulin receptor (IR) in intrinsic/adaptive resistance to anti-IGF-1R therapies^7^,^8^, (2) counterbalance of the antiangiogenic effects of IGF-1R inhibitors via IGF-2 production^33^ and (3) IGF-induced integrin-Src signalling module as a resistance mechanism against anti-IGF-1R monoclonal antibody-based anticancer therapies^34^, we hypothesized that the IGF axis could have contributed to the stromal cell recruitment. As shown by the quantitative real-time PCR and western blot analysis, IGF-1, IGF-1R and IR expression were not increased in the cixutumumab-treated cancer and stromal cells (Fig. 5a and Supplementary Fig. 11), but cixutumumab treatment induced a significant transcription-dependent IGF-2 production in cancer cells, as shown by quantitative real-time PCR (Fig. 5a) and western blot (Fig. 5b) analyses. Luciferase analysis also showed greater levels of activity of IGF-2 promoters^35^,^36^ in the cixutumumab-treated cells than in vehicle-treated cells (Supplementary Fig. 12). Immunofluorescence staining of cancer cells treated with cixutumumab revealed an increased level of IGF-2 production (Fig. 5c). Moreover, IGF-1R shRNA-transfected H1299 cells also revealed transcriptional increases in IGF-2 expression (Fig. 5d). In contrast, IGF-2 expression was either not detectable in Wi38, MRC5 and THP-1 cells (Fig. 5e) or only modestly induced by the cixutumumab treatment in HUVECs (Fig. 5a). Interestingly, Wi38, MRC-5 and THP-1 cells exhibited prominent IGF-2R expression when compared with HUVECs (Fig. 5e). These expression patterns are reminiscent of fibroblast and monocyte cell line migration but not of VE cells towards CM from cixutumumab-treated cancer cells (Fig. 4). We therefore assessed the role of IGF-2 and IGF-2R in cancer–stroma communication. Cixutumumab treatment induced a minimal change in the proliferation of H1299 cells with an shRNA-mediated loss of IGF-2 expression (Supplementary Fig. 13). In contrast, CM from the H1299 cells without any IGF-2 expression was significantly less effective at inducing Wi38 and THP-1 cell migration than the CM from the control cells (Fig. 5f). Moreover, an shRNA-induced knockdown of IGF-2R expression (Fig. 5g, left) significantly suppressed the migration of Wi38 and THP-1 cells towards the CM from cixutumumab-treated H1299 cells (Fig. 5g, right). Therefore, these findings indicate that intercellular IGF-2/IGF-2R interactions mediate the recruitment of stromal cells by cancer cells during IGF-1R blockade.

### Cixutumumab activates STAT3 leading to IGF-2 transcription

**Figure 6:**
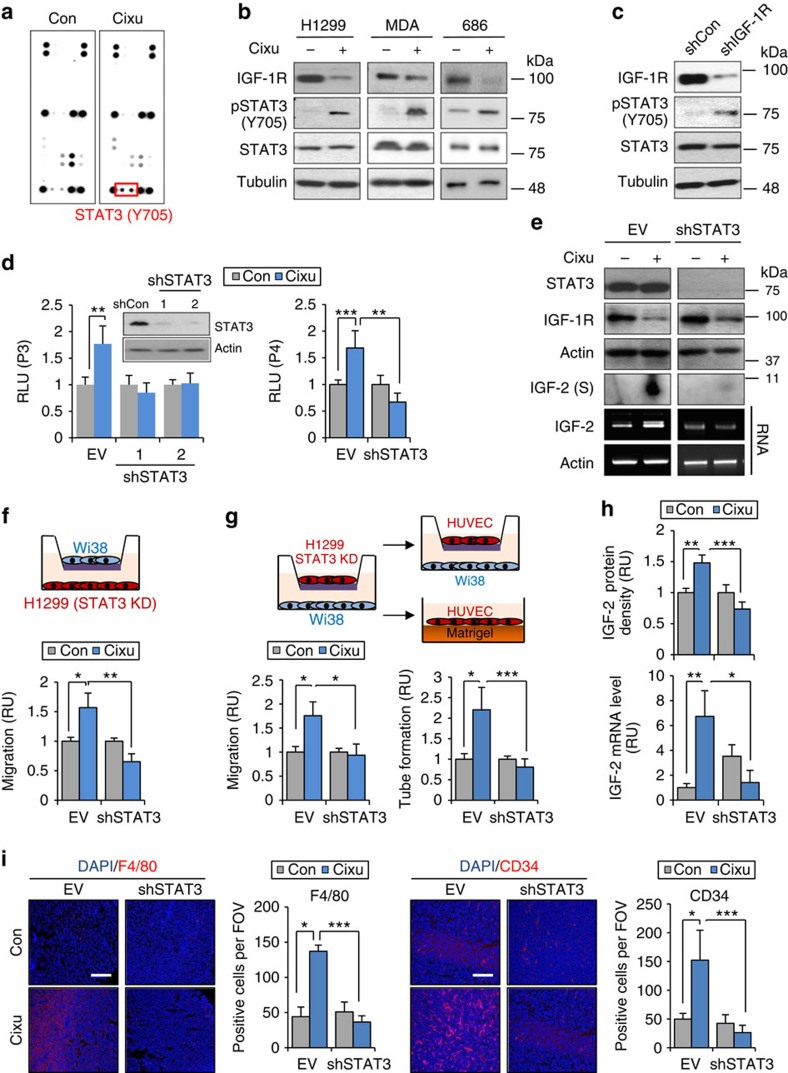
IGF-1R blockade-induced IGF2 transcription via STAT3 activation. (**a**) Protein lysates from cixutumumab-treated H1299 cells were incubated with phospho-RTK arrays. Spots are in duplicate, with each pair corresponding to a specific pRTK. The pair spots in the corners are positive controls. STAT3 phosphorylation was determined by western blotting in (**b**) cixutumumab-treated or (**c**) IGF-1R-silenced H1299 cells. (**d**) Luciferase assay for IGF-2 promoter activity. Insert: STAT3 expression was reduced by shRNA in H1299 cells as confirmed by western blotting. Left: P3 promoter. Right: P4 promoter. Each bar represents the mean relative luciferase unit (RLU) ±s.d. of four identical wells of a single representative experiment. (**e**) The effect of cixutumumab on IGF-2 expression in H1299 cells with reduced STAT3 expression was determined by RT–PCR and western blotting. (**f**) Wi38 cells were seeded in the top chamber of the transwell insert. Cixutumumab-treated H1299 cells transfected with empty vector or shSTAT3 expression were seeded in the bottom chambers of the transwell. Wi38 cells were allowed to migrate for 16 h. Each bar represents the mean relative unit (RU) ±s.d. of three replicates of a single representative experiment. (**g**, left) Empty vector- or shSTAT3-transfected H1299 cells were treated with cixutumumab and co-cultured with Wi38 cells in the transwell. After 24 h, HUVEC cells were seeded in the new top-chamber insert and cell migration was analysed after 6 h. (**g**, right) HUVEC cells were seeded onto Matrigel and incubated with media from Wi38 cells for 10 h. Each bar represents the mean relative unit (RU) ±s.d. of three replicates of a single representative experiment. (**h**,**i**) MDA231 cells stably transfected with either an empty vector or an expression carrying shSTAT3 were mixed with Wi38 cells (ratio 2:1), orthotopically injected into BALB/c-nude mice (*n*=3–5 per group) and treated with cixutumumab (10 mg kg^−1^, intraperitoneally, once weekly) for 5 weeks. (**h**) IGF-2 expression in the excised primary tumours was assessed by real-time PCR (bottom) and densitometric quantification of western blotting (top). Data are presented as mean densitometric quantification of western blotting or mRNA level ±s.d. (**i**) The excised primary tumours were assessed by immunofluorescence staining using anti-F4/80 (left) and -CD34 (right) antibodies (Scale bar, 50 μm). Data are presented as mean positive cells per field of view (FOV) ±s.d. *P<0.05, ** *P*<0.01 and ***P<0.001 by two-sided Student’s *t*-test.Cixu, cixutumumab (25 μg ml^−1^, 6 days for cell treatment); Con, control; EV, empty vector.

We investigated the mechanisms that mediate the cixutumumab-induced IGF-2 transcription. By using a human phospho-receptor tyrosine kinase (RTK) signalling array kit, it was shown that there was a remarkably increased STAT3 phosphorylation in cixutumumab-treated H1299 cells when compared with the control cells (Fig. 6a). When compared with the control cells as well, the indicated cixutumumab-treated (Fig. 6b) or IGF-1R-silenced cancer (Fig. 6c) cells showed increased levels of STAT3 phosphorylation, which was established by western blotting. With the cixutumumab-induced activation of integrin/Src^34^ and the capacity of Src to activate STAT3 (ref. 37), STAT3 activation during an IGF-1R blockade could have been mediated by integrin/Src signalling. Indeed, the cancer cells treated with cixutumumab or IGF-1R shRNA revealed FAK phosphorylation at tyrosine 397, a residue phosphorylated by integrin clustering^38^ (Supplementary Fig. 14). Luciferase reporter, western blotting and reverse transcription–PCR (RT–PCR) analyses revealed that cixutumumab-induced IGF-2 promoter activation (Fig. 6d) and expression (Fig. 6e) were abrogated in H1299 cells in which STAT3 expression had been silenced by shRNA transfection. In addition, the STAT3-knocked down H1299 cells revealed a significantly decreased ability to recruit Wi38 cells (Fig. 6f) and to mediate Wi38 cells’ stimulation of HUVEC migration and tube formation (Fig. 6g) on cixutumumab treatment. We further evaluated the effects of STAT3 knockdown on cancer–stroma interaction *in vivo*. Cixutumumab-treated tumours of STAT3 shRNA-transfected MDA231–Luc cells showed significant decreases in levels of IGF-2 expression (Fig. 6h), F4/80^+^ macrophages (Fig. 6i, left) and CD34^+^ VE cells (Fig. 6i, right) when compared with control tumours. These results suggest that STAT3 plays a key role in cixutumumab-induced IGF-2 transcription, stromal cell infiltration and tumour angiogenesis.

### CXCL8 production in the TME stimulates angiogenesis

**Figure 7:**
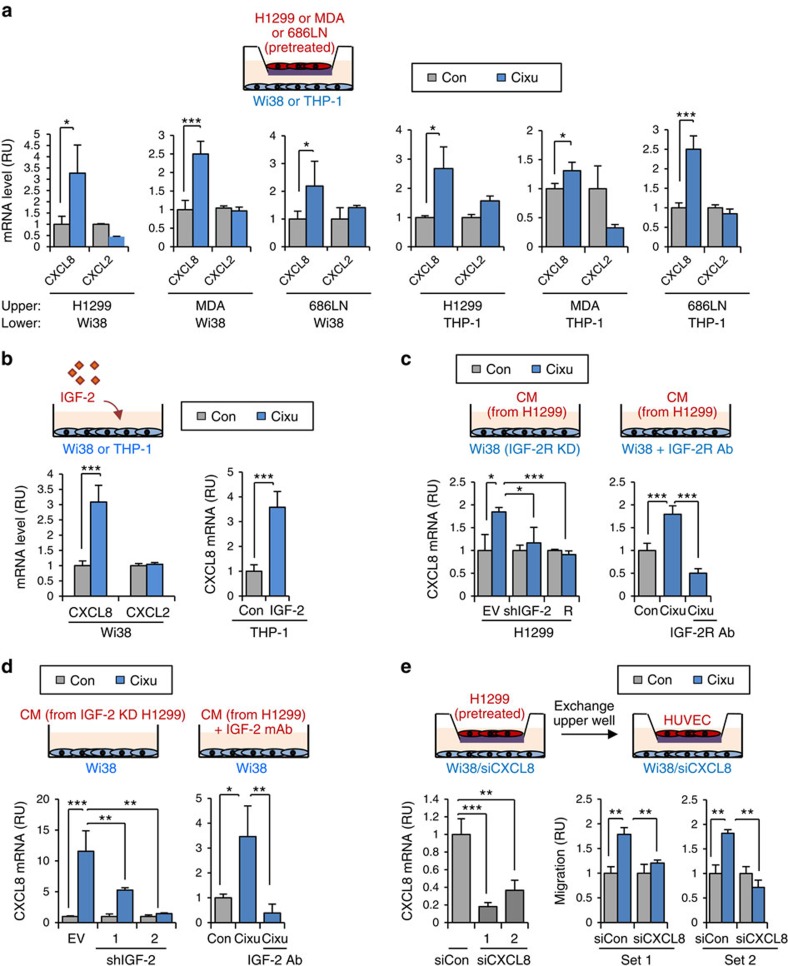
Increased CXCL8 production from stromal cells through the IGF-2/IGF-2R interaction. (**a**) Wi38 or THP-1 cells were co-cultured with cixutumumab-treated cancer cells in the transwell for 24 h and the mRNA level of each target was determined by real-time PCR. (**b**) Wi38 or THP-1 cells were treated with rhIGF-2 (100 ng ml^−1^, 24 h) and CXCL8 mRNA levels were examined by real-time PCR. (**c**) IGF-2R expression was reduced by shRNA (left) or anti-IGF-2R-neutralizing antibody (10 μg ml^−1^) (right) in Wi38 cells and followed by treatment with CM from cixutumumab-treated cancer cells for 24 h. CXCL8 mRNA levels were determined by real-time PCR. (**d**) Wi38 cells were incubated with CM from cixutumumab-treated H1299 transfected with empty vector or shIGF-2 (left) or anti-IGF-2-neutralizing antibody (5 μg ml^−1^) and CM from cixutumumab-treated H1299 cells (right). After 24 h, CXCL8 mRNA levels were examined by real-time PCR. (**e**, left) Wi38 cells were transfected with negative control siRNA or siCXCL8 and knockdown of CXCL8 was confirmed by real-time PCR. (**e**, right) Transfected with each siRNA were seeded in the bottom chamber and co-cultured with cixutumumab-treated H1299 cells in the transwell. After 24 h, the top chambers were removed and HUVEC cells were seeded in the new top chamber. HUVEC cells were allowed to migrate for 6 h. *P<0.05, **P<0.01 and ***P<0.001 by two-sided Student’s *t*-test. Cixu, cixutumumab (25 μg ml^−1^, 6 days for cell treatment); Con, control; EV, empty vector; RU, relative unit.

Our next step was to determine whether infiltrated stromal cells secrete soluble factors to recruit VE cells by analysing Wi38 and THP-1 cells for the expression of key cytokines involved in tumour-promoting microenvironments^39^. We observed consistent increases in CXCL8/IL8 transcripts in Wi38 and THP-1 cells treated with CM from cixutumumab-treated H1299, MDA231 or 686LN cells (Supplementary Fig. 15), or those co-cultured with cixutumumab-pretreated H1299, MDA231 or 686LN cells (Fig. 7a). The small cytokine CXCL8 is one of the most potent chemoattractant and proangiogenic factors^40^,^41^,^42^,^43^, and exhibits a wide range of actions on various types of cells including VE cells^28^,^44^,^45^. It has been demonstrated that HUVECs express CXCR1 and CXCR2, and respond to CXCL8 (refs 46, 47). We also confirmed the HUVECs’ responsiveness to CXCL8 by migration and tube formation assays (Supplementary Fig. 16). An enzyme-linked immunosorbent assay (ELISA) confirmed the secretion of CXCL8 from Wi38 cells that were co-cultured with cixutumumab-treated MDA231 cells (Supplementary Fig. 17). Wi38 and THP-1 cells also exhibited increased CXCL8 messenger RNA after stimulation with rhIGF-2 (Fig. 7b). The CM from cixutumumab-treated cancer cells failed to increase CXCL8 transcripts in Wi38 cells with an shRNA-mediated loss of IGF-2R expression and also in cells that had been incubated with neutralizing antibody blocking IGF-2R (Fig. 7c and Supplementary Fig. 18). This corresponded with the CM from H1299 cells with a reduced IGF-2 expression, which failed to stimulate CXCL8 transcription in Wi38 cells. Incubation with anti-IGF-2-neutralizing monoclonal antibody also blocked the ability of Wi38 cells to induce CXCL8 transcription in response to the CM from cixutumumab-treated H1299 cells (Fig. 7d). Therefore, we then assessed whether CXCL8 released from stromal cells plays a functional role in stimulating angiogenesis. Wi38 cells incubated with cixutumumab-treated H1299 cells significantly increased HUVEC migration when compared with control cells (Fig. 7e). When CXCL8 expression in Wi38 cells was reduced by small interfering RNA (siRNA), the ability of Wi38 cells to induce HUVEC migration was significantly decreased. These findings suggested that macrophage- and fibroblast-induced CXCL8 secretion via communication between cancer and stromal cells may induce the recruitment of endothelial cells, leading to vascular formation.

### Changes in TME under cixutumumab clinical trial

**Figure 8:**
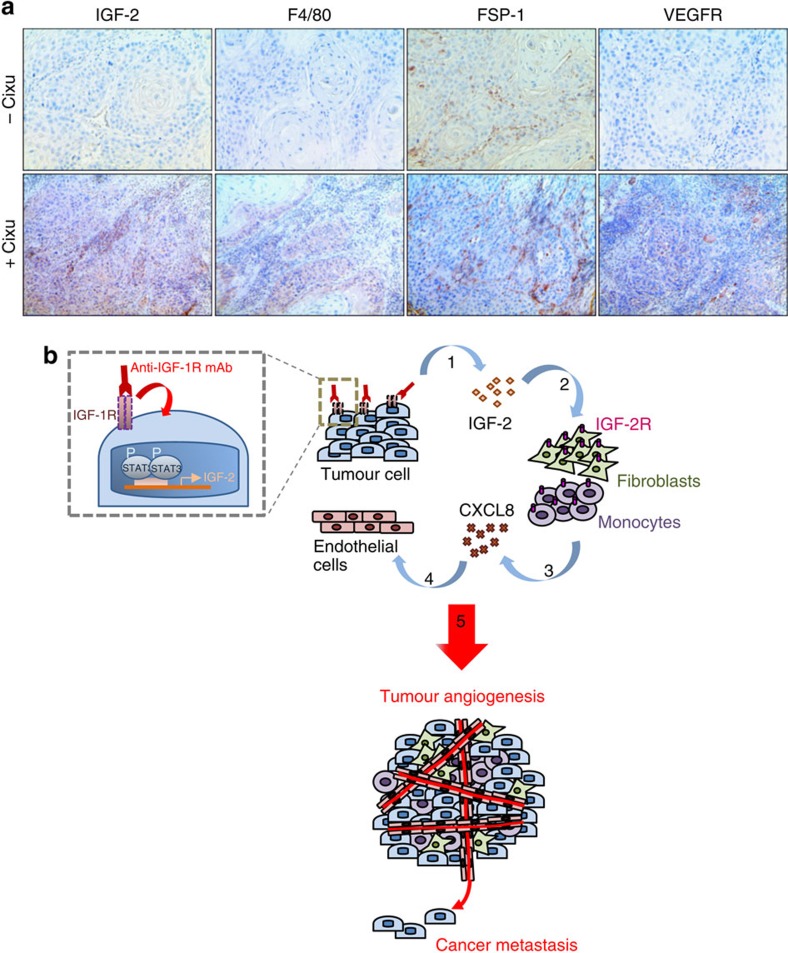
Clinical relevance of tumour-associated macrophages and fibroblasts in HNSCC patients with cixutumumab treatment. (**a**) Representative images of IHC analyses on IGF2 expression, VEGFR^+^ VE cells, F4/80^+^ macrophages and FSP-1^+^ fibroblasts in the tissue samples from patient with HNSCC, either naive or treated with cixutumumab for 3 weeks. Images are from patient 1 of each group. Other images are included in Supplementary Fig. 19. (**b**) Schematic model of events noted in the TME on treatment with IGF-1R-targeted therapy. IGF-1R blockade by monoclonal antibodies (mAbs) in cancer cells leads to STAT3 activation, resulting in IGF-2 production (1). IGF-2 secreted from cixutumumab-treated cancer cells recruits monocytes and fibroblasts in the TME through IGF-2/IGF-2R interaction (2). Recruited stromal cells stimulated the production of the proangiogenic cytokine, CXCL8 (3). CXCL8 attracts endothelial cells (4). Increased tumour angiogenesis on IGF-1R blockade promotes cancer metastasis (5).

We investigated whether our findings up to this point can be practically applied to understanding the mechanisms of cixutumumab resistance in human patients. To this end, we analysed F4/80^+^ macrophages, FSP-1^+^ fibroblasts and VEGFR^+^ VE cells along with the IGF-2 expression in HNSCC tissues resected from patients (*n*=6) who enroled in a clinical trial with cixutumumab. Compared with HNSCC tissues (*n*=10) from head and neck cancer tissue array (US Biomax Inc., Rockville, MD), all six samples from cixutumumab-treated patients showed markedly increased numbers of macrophages, fibroblasts and VE cells along with IGF-2 expression (Fig. 8a and Supplementary Fig. 19). Although additional studies using a larger number of cases are required, these findings suggest that the cixutumumab-induced increases in tumour-associated macrophages and fibroblasts may play a role in IGF-2 expression and predict resistance to IGF-1R monoclonal antibody-based therapies in cancer patients.

## Discussion

Molecularly targeted therapies have raised the hope of developing more effective therapeutic strategies with reduced side effects. However, in many cases, targeted drugs exhibit only temporary efficacy and resistance eventually emerges. Resistance to anticancer drugs has most often been attributed to cell-autonomous mechanisms of drug resistance via genetic or epigenetic changes in tumour cells. However, recent studies have focused on the role of the TME in innate resistance to anticancer drugs^13^,^14^,^48^,^49^,^50^,^51^,^52^. The studies reported herein suggest that IGF-1R-targeted approaches create a milieu that stimulates tumour angiogenesis, thus accelerating cancer metastasis and promoting a tumour’s adaptive–evasive response to the therapy. Our study demonstrates the following findings (Fig. 8b): (1) IGF-1R blockade by treatment with anti-IGF-1R monoclonal antibody cixutumumab reprogrammes cancer cells to activate STAT3, leading to transcriptional upregulation of IGF-2; (2) IGF-2 acts as a chemoattractant to monocytes and fibroblasts via utilization of IGF-2R on stromal cells; and (3) infiltrated stromal cells produce various proangiogenic cytokines, most notably CXCL8, which act as chemoattractants to VE cells, resulting in tumour angiogenesis and thus facilitating cancer metastasis. Our results offer mechanistic insights into the interplay between tumours and their healthy counterparts within the TME in the development of adaptive–evasive abilities in the face of an IGF-1R blockade.

Although IGF-1R signalling is a validated target in solid tumours, ultimate disease progression in the face of IGF-1R-targeted therapies has been described in clinical trials^2^. In our study, anti-IGF-1R treatment in mice bearing orthotopic tumours of human cancer cells induced a period of stable disease followed by spontaneous metastasis and a reduced life span, which is debatably similar to the observations in clinical trials with figitumumab in combination with chemotherapy^53^. This escape is not a failure in the IGF-1R blockade, because IGF-1R dephosphorylation was confirmed after short-term or prolonged drug treatment *in vitro* and *in vivo*. In respect to the mechanisms of resistance to anti-IGF-1R therapies, several studies have implicated cross-talk via alternative epithelial RTKs such as EGFR, IR, platelet-derived growth factor-β and HER2 (refs 7, 8, 9, 10) or non-receptor transmembrane signallers such as integrins^34^ in cancer cells. However, in our study, cancer cells subjected to an IGF-1R blockade appeared to have the ability to recruit macrophages and fibroblasts, which subsequently recruited VE cells. These observations were consistent with the well-established importance of communication among various cell types within the TME in anticancer drug resistance^48^,^54^. Therefore, resistance to anti-IGF-1R therapy is not only likely to be due to activities in the tumour cells but also due to the complex interplay between tumour cells and their neighbouring heterologous stromal cells within the TME.

The interaction between tumours and stromal cells in the TME is mediated through cell adhesion molecules and/or soluble factors^49^. For example, CCL2 secreted from both tumour and stromal cells was reported to mediate interaction between inflammatory monocytes and tumour cells^48^. IGF-2 levels have been implicated in drug resistance in anti-IGF-1R inhibitors^7^ and anti-EGFR monoclonal antibody^55^. We also observed transcriptional upregulation of IGF2 in cancer cells after IGF-1R blockade by monoclonal antibody. Our evolving data identify the importance of the intercellular IGF-2/IGF-2R system in cancer cell communication with the TME showing that: (1) macrophages and fibroblasts but not ECs migrate towards cixutumumab-treated cancer cells or CM obtained from the cancer cells; (2) IGF-2R expression is observed in macrophages and fibroblasts but not in HUVECs; and (3) the ability of cancer cells to chemoattract stromal cells is inhibited by silencing IGF-2 or IGF-2R expression in cancer cells or stromal cells, respectively. Our subsequent study identified that upregulated IGF-2 expression is primarily, if not solely, a result of cixutumumab-induced STAT3 activation.

We further demonstrated that the small cytokine CXCL8, which serves as a shared ligand of CXCR1 and CXCR2 (ref. 56), is significantly upregulated in macrophages and fibroblasts in response to the IGF-2/IGF-2R interaction, subsequently inducing VE cell recruitment. Although IGF-2R is a scavenger receptor that regulates IGF-2 levels by endocytosis-mediated degradation^2^, the action of IGF-2 through IGF-2R transmembrane signalling has also been demonstrated in previous studies^57^,^58^,^59^,^60^,^61^,^62^,^63^. CXCL8 is one of the major activators of the PI3K/Akt and ERK pathways^64^ and acts as a potent proangiogenic chemokine and chemoattractant for a wide range of cells, including endothelial cells^40^,^41^,^42^,^65^. In addition, CXCL8 is involved in the initiation of tumour-associated inflammation, neovascularization and tumour progression^43^,^66^. Therefore, increased CXCL8 production in the TME potentially contributes to tumour angiogenesis and cancer progression. Thus, an attractive hypothesis is that increased epithelial IGF-2 production leads to macrophage and fibroblast chemotaxis, which subsequently stimulates CXCL8 expression in stromal cells through the IG-F2/IGF-2R axis, resulting in endothelial cell recruitment and cancer cell migration. In support of the supposition, our data using paraffin-embedded tissues from HNSCC patients in a cixutumumab clinical trial showed significant increases in tumour-associated macrophages, cancer-associated fibroblasts, VE cells and IGF-2 expression. Given that the downstream intracellular targets of CXCL8 include STAT3, increased CXCL8 in the tumour stroma promotes a positive intercellular feedback loop between CXCL8 and IGF-2 production. Previous studies have suggested that tumour-derived IGF-2 contributes to vasculogenesis by augmenting the recruitment of endothelial progenitor cells through its interaction with IGF-2R^57^. Therefore, IGF-2 secretion could have induced mobilization of endothelial progenitor cells and bone marrow derived progenitor cells to the TME, leading to an increased vasculature in cixutumumab-treated xenograft tumours.

In conclusion, our data identify anti-IGF-1R monoclonal antibody-induced cancer–stromal cell communication via STAT3-dependent IGF-2 production in cancer cells and a positive IGF-2/CXCL8 feedback loop through the intercellular IGF-2/IGF-2R system. Although additional validation studies using corroborated clinical samples from IGF-1R-targeted therapies must be performed, our current study reveals malignant progression of tumours in the face of an IGF-1R blockade through IGF-2 secretion in the TME. Given that several approaches to developing anti-IGF-2 monoclonal antibody and STAT3 inhibitors for cancer treatment are ongoing in clinical trials^67^, our data will have a clinical impact. Clinical trials are warranted to assess whether therapeutic strategies targeting STAT3 or IGF-2 would circumvent tumour angiogenesis and pro-invasive consequences, overcoming innate resistance to anti-IGF-1R monoclonal antibody-based therapies.

## Methods

### Cell culture and specimens

Human HNSCC cell line (686LN) was kindly provided by Dr Jeffrey Myers (MD Anderson Cancer Center, Houston, TX). In our study, we used MDA-686LN, which is on the iclac list of misidentified cell lines, as a representative head and neck cancer cell line. Our study is not restricted to recurrence or lymph node metastasis. Hence, misidentification of MDA686LN will not affect our conclusion. Human NSCLC cell line (H1299) was purchased from the American Type Culture Collection (Manassas, VA, USA). MDA231–Luc cell line was obtained from Caliper Life Science (Alameda, CA, USA). Human fibroblast Wi38 cells were kindly provided by Dr John V. Heymach (MD Anderson Cancer Center) and MRC-5 cells were purchased from the Korean Cell Line Bank (Seoul, Republic of Korea). Human monocyte THP-1 cells were kindly provided by Dr Kyu-Won Kim (Seoul National University, Seoul, Korea). Mouse endothelial cells (SVECs) and mouse breast cancer cell line (4T1) were kindly provided by Dr Mien-Chie Hung (MD Anderson Cancer Center). Cells were cultured in DMEM, DMEM/F-12 or RPMI1640 medium supplemented with 10% fetal bovine serum. HUVEC cells were purchased from Invitrogen (Grand Island, NY, USA) and grown in vasculife basal medium supplemented with vasculife VEGF life factors (Lifeline Cell Technology, Frederick, MD, USA). Cells were kept in 37 °C with 5% CO_2_. The cells were free of mycoplasma contamination.

Cixutumumab (IMC-A12), a fully human IgG1 monoclonal antibody, was provided by ImClone Systems (New York, NY, USA). Human recombinant IGF-2, human recombinant CXCL8 and neutralizing antibodies against the IGF-2 or IGF-2R were obtained from R&D Systems (Minneapolis, MN, USA). Transfection with expression vector, siRNA or shRNA vectors was performed using Lipofectamine 2000 (Invitrogen) or Fugene 6 (Promega, Madison, WI, USA). siRNA for negative control 1 (sense, 5′- UUCUCCGAACGUGUCACGUTT -3′, antisense, 5′- ACGUGACACGUUCGGAGAATT -3′) and CXCL8 (1: sense, 5′- GAAGAGGGCUGAGAAUUCATT -3′, antisense, 5′- UGAAUUCUCAGCCCUCUUCTT -3′; 2: sense, 5′- GCCAGAUGCAAUACAAGAUTT -3′, antisense, 5′- AUCUUGUAUUGCAUCUGGCTT -3′) were purchased from GenePharma (Shanghai, China), and negative control 2 were purchased from Bioneer (South Korea). Matrigel for mouse experiment, migration assay and tube formation assay was obtained from BD Biosciences (San Jose, CA, USA). All other materials were purchased from Sigma-Aldrich (St Louis, MO, USA). Head and neck cancer tissue array HN242a was purchased from US Biomax Inc.. Cixutumumab-treated cancer tissues were obtained from Department of Thoracic/Head and Neck Medical Oncology, The University of Texas M.D. Anderson Cancer Center.

### Mouse studies

All mouse study procedures were performed in accordance with protocols approved by the Institutional Animal Care and Use Committee of Seoul National University (SNU-130705-2-2 and SNU-141217-9). Mice were cared for in accordance with guidelines set by the Association for Assessment and Accreditation of Laboratory Animal Care and the US Public Health Service Policy on Human Care and Use of Laboratory Animals. For orthotopic tumour models, MDA231 (2 × 10^5^ cells per mouse in 40 μl containing Matrigel), H1299 (1 × 10^6^ cells per mouse in 100 μl of PBS) and 686LN (2 × 10^5^ cells per mouse in 40 μl containing Matrigel) were injected into 6-week-old female nude (H1299, 686LN and MDA 231; Fig. 1, first group) or 6-week-old female NOD/SCID (MDA231; Fig. 1, second and third group) mice at mammary fat pad, lung or tongue sites. When the solid tumours of MDA231 reached to a volume of 100 mm^3^, mice were treated with cixutumumab (10 mg kg^−1^, intraperitoneally, once weekly). For orthotopic tumour models of H1299 or 686LN, mice were treated with cixutumumab 1 week or 3 days after the orthotopic injection, respectively. Tumour volume was determined by measuring the short and long diameters of the tumours with a caliper. The tumour volume was calculated using the following formula: tumour volume (mm^3^)=(short diameter)^2^ × (long diameter) × 0.5. Mice were killed when tumours reached >10% of body weight or mice became moribund. Tumour volume was measured every 2 days. To establish MDA231 orthotopic tumours in mice, in which the human lymphoid system is reconstituted, the NOD/SCID/JAK3^null^ mouse model^22^ was employed. Human cord blood was obtained from normal full-term deliveries. Informed consents were obtained according to Institute guidelines and these works were approved by Seoul Metropolitan Government Seoul National University Boramae Medical Center (IRB No. 16-2014-80) and Seoul National University (IRB No. E1409/002-001) Institutional Review Board. Briefly, NOD/SCID/Jak3^null^ mice were preconditioned with busulfan (30 mg per kg body weight) and then human CD34+ haematopoietic stem cells purified from human cord blood were transplanted. Peripheral blood from the retro-orbital sinus and the spleen samples obtained at 4 weeks after transplantation or at the time of killing, respectively, were analysed by flow cytometry, for the presence of human cells expressing the CD45 human leukocyte antigen. Fluorescein isothiocyanate- or allophycocyanin-conjugated monoclonal antibodies against human or mouse CD45 (eBioscience, San Diego, CA) were added to splenocytes or peripheral blood mononuclear cells, incubated for 30 min at 4 °C, washed twice and analysed using a FACS Calibur flow cytometer (BD Biosciences). For orthotopic tumour models from MDA231 cells with reduced expression of STAT3, mixture of cancer cells and Wi38 fibroblast cells (1 × 10^6^ cells: 5 × 10^5^ cells per mouse in 30 μl containing Matrigel) were injected into nude mice at mammary fat pad.

### Immunofluorescence and IHC assays

Cells were grown on coverslips and fixed in methanol, blocked with 3% BSA solution, incubated with primary antibodies (IGF-2, F-20, Santa Cruz Biotechnology, Santa Cruz, CA, USA; 1:200 dilution) and incubated with fluorescence-conjugated secondary antibodies (Alexa 488-conjugated goat antibody, Invitrogen; 1:1,000 dilution). Samples were counterstained with 10 μg ml^−1^ DAPI (4’,6-diamidino-2-phenylindole) to detect all nuclei. For immunostaining of tumour tissue, mice were anaesthetized by intraperitoneal injection of Zoletil (Virbac) and Rompun (Bayer), and tumour tissues were dissected and embedded in optimum cutting temperature compound (Sakura) or fixed with paraformaldehyde and embedded in paraffin. For immunofluorescent staining, 4 μm frozen sections were fixed in 4% paraformaldehyde for 30 min, permeabilized with 0.3% Triton X-100 for 15 min and blocked in protein block solution (Dako) for 30 min. Primary antibodies (CD34, MEC 14.7, Santa Cruz Biotechnology; IGF-2, F-20, Santa Cruz Biotechnology; F4/80, CI:A3-1, Serotec; 1:100 dilution) were diluted in 3% BSA solution and incubated overnight at 4 °C. Then, samples were washed twice with 1 × PBS containing 0.05% Tween-20 (PBST) and incubated with fluorescence-conjugated secondary antibodies (Alexa 488-conjugated goat antibody and Alexa 594-conjugated rat antibody, Invitrogen; 1:1,000 dilution) for 1 h. Next, samples were counterstained with DAPI. For immunohistochemistry, 4 μm paraffin sections were rehydrated, blocked and incubated with antigen retrieval buffer (Vector Laboratories) at 95 °C for 20 min. Samples were incubated with primary antibodies (Luciferase, Abcam; CD45, IBL-3/16, Serotec; F4/80, CI:A3-1, Serotec; F4/80, Thermo; FSP-1, Abcam; Iba-1, Wako; VEGFR1, Y103, Abcam; human mitochondrion, Abcam; 1:100 dilution in 3% BSA solution) overnight at 4 °C, washed twice with PBST and incubated with appropriate biotinylated secondary antibodies for 1 h at room temperature. Staining were revealed using Diaminobenzidine substrate kit (Vector Laboratories). Slides were counterstained with haematoxylin and examined using Nuance fluorescence microscope (Perkin Elmer).

### Establishment of silenced stable cell line

For stable knockdown cell line establishment, shRNA bacterial glycerol stock complementary to each human gene-coding sequences (IGF-2, NM_000612; IGF-1R, NM_000875.2; IGF-2R, NM_000876; and STAT3, NM_003150) were purchased (Mission shRNA, Sigma) and the DNA construct (in PLKO.1 lentiviral vector backbone) was isolated from bacterial culture. Lentiviral production was performed by transfection of HEK293T cells using Fugene 6 (Promega). Supernatants were collected 24–48 h after transfection and then filtered through 0.22-μm syringe filter. Cells were infected with lentivirus with 8 μg ml^−1^ polybrene for 24 h and then medium was replaced with fresh growth media containing puromycin (1–2 μg ml^−1^) for selection.

### Isolation of primary monocytes

Primary cells were isolated from 686LN xenografted tumours. After the dissection of tumours, single cells were isolated using tumour tissue dissociation kit (Miltenyi Biotech) and then initially positively sorted using CD11b microbeads using a MACS seperator and MS columns (Miltenyi Biotech). CD11b^+^ population were further sorted using a FACS Aria cells were isolated using magnetic beads (Miltenyi Biotech). Macrophages were sorted using FACS Aria (BD Biosciences). Cells were labelled with fluorochrome-conjugated antibodies, PE/Cy7 anti-mouse Ly-6G and PerCP/Cy5.5 anti-mouse CD11b (Biolegend).

### *In vitro* migration and tube formation assay

For transwell (8.0 μm pore size, Corning) migration assay, the outer membrane was coated with 0.05% gelatin. The indicated cells (1 × 10^5^ cells for THP-1 cells and 4 × 10^4^ cells for other cells) were seeded onto the upper wells and CM from cancer cells or NIH3T3 cells were used as chemoattractant. Cells were incubated for 12–20 h and the incubation time was dependent on types of the cell lines. After the incubation, membrane was stained with haematoxylin solution and mounted onto the slide glass. The number of stained cells per field was counted using a microscope at × 100 magnification. CM was collected from cells that had been treated with cixutumumab for 6 days and medium was exchanged with serum-free medium for 24 h. CM was concentrated using the Amicon Ultra-4 centrifugal filter device. For tube formation, 96-well plate was coated with Matrigel (BD Bioscience) then HUVEC (1 × 10^4^ cells per well) cells with CM was seeded and incubated. Tube formation was checked after 6–10 h using a microscope. Three independent experiments were performed with similar results.

### RT–PCR and real-time PCR analysis

Total RNA was isolated from cells or mice using TRIzol reagent (Invitrogen) and transcribed using a PrimeScript 1st Strand cDNA Synthesis Kit (Takara, Otsu, Shiga, Japan) according to the manufacturers’ protocols. For RT–PCR, complementary DNA was amplified using gene-specific primer sets (Supplementary Table 1) with EconoTaq 2 × Master Mix (Lucigen, Middleton, WI, USA). PCR products were identified using electrophoresis on 1.5 % agarose gels containing RedSafe (Intron, South Korea). Quantitative real-time PCR was performed in triplicate on Light Cycler 480 (Roche Diagnostics, Indianapolis, IN, USA) using Light Cycler SYBR Green I Master (Roche) and data were analysed on the basis of threshold cycle values of each sample and normalized with β-actin. Primer sets used in real-time PCR are shown in Supplementary Table 2.

### Western blotting and RTK array

Cells were collected with RIPA lysis buffer (50 mM Tris-HCl pH 7.4, 150 mM NaCl, 1% NP-40, 0.25% sodium deoxycholate, 1 mM EDTA), containing 1 mM Na_3_VO_4_, 100 mM NaF, 10 mM NaPP and protease inhibitor cocktail (Roche). Protein lysates were recovered by centrifugation at 13,000 r.p.m. at 4 °C and then protein concentrations were determined by the BCA assay kit (Thermo Scientific, Rockford, IL, USA). Proteins were separated by SDS–PAGE and transferred to polyvinylidene difluoride membranes (Bio-Rad, Hercules, CA, USA). The membranes were incubated with primary antibodies against IGF-1 (H-70, 1:500), IGF-2 (F-20, 1:500), IGF-1R (C-20, 1:1,000), IGF-2R (H-300, 1:1,000), actin (C-11, 1:2,000), IR (C-19, 1:1,000) (Santa Cruz Biotechnology), tubulin (9F3, 1:2,000), STAT3 (79D7, 1:2,000), phospho-STAT3 (Y705) (D3A7, 1:1,000) (Cell Signaling Technology, Denvers, MA, USA), FAK (77, 1:1,000, BD) and phosphor-FAK (Y397) (EP2160Y, 1:1,000, Millipore, Billerica, MA, USA). After removing the primary antibody, membranes were incubated with horseradish peroxidase-conjugated secondary antibodies. The signals were visualized using SuperSignal West Femto Chemiluminsecent Substrate (Thermo Scientific). Full blottings are shown in Supplementary Fig. 20. For RTK signalling array, H1299 cells were treated with 25 μg ml^−1^ cixutumumab for 6 days, followed by assay using PathScan RTK Signaling Antibody Array Kit (Cell Signaling Technology) according to the manufacturer’s protocols.

### Enzyme-linked immunosorbent assay

ELISA assay was performed by coating 96-well plates with 1 mg per well of anti-IL-8 (R&D Systems). Before the subsequent steps in the assay, the coated plates were washed twice with PBST. All reagents and coated wells used in this assay were incubated for 2 h at room temperature. Following exposure to the medium, the assay plates were exposed sequentially to each of the biotin-conjugated secondary antibodies, as well as AP and ABTS substrate solution containing 30% H_2_O_2_. The plates were read at an absorbance of 405 nm. Appropriate specificity controls were included and all samples were run in duplicate.

### Plasmids and luciferase assay

pGL3-IGF-2 promoter reporter plasmids (P3) were kindly provided by Dr P. Elly Holthuizen (Utrecht University, Utrecth, The Netherlands). IGF-2 promoter 4 region was inserted into the XhoI–HindIII restriction site of the pGL3 basic reporter vector (Promega). pRL-Tk *Renilla* reporter plasmid was purchased from Promega. Cells pretreated with cixutumumab for 6 days were seeded into 24 wells and then transfected with each reporter vectors and pRL-Tk vectors. Forty-eight hours after transfection, luciferase activity was measured using Dual-Glo Luciferase Assay System (Promega). Transfection efficiency was determined by normalizing the reporter activity with *Renilla* luciferase activity.

### Cell proliferation/viability assay

Cells (8 × 10^3^) were seeded into 24-well dishes and cell numbers were assessed using a haemocytometer on days 2, 4 and 6. Four replicate wells were used for each analysis. For cell viability assay, cells were seeded into 96-well dish and treated with cixutumumab (25 μg ml^−1^) for various time. Viability of THP-1 cells were measured by MTS (3-(4,5-dimethylthiazol-2-yl)-5-(3-carboxymethoxy phenyl)-2-(4-sulfophenyl)-2H-tetrazolium) assay. Viability of other cells were measured by MTT (3-(4,5-dimethylthiazol-2-yl)-2,5-diphenyltetrazolium bromide) assay. Three independent experiments were performed with similar results.

### Statistical analysis

Statistical comparisons between two groups were performed with unpaired Student’s *t*-test and two-sided *P*-value of <0.05 was considered statistically significant. For survival curve analysis, two-tailed *P*-values were calculated using the Mantel–Cox log-rank test.

## Additional information

**How to cite this article:** Lee J.-S. *et al*. STAT3-mediated IGF-2 secretion in the tumour microenvironment elicits innate resistance to anti-IGF-1R antibody. *Nat. Commun.* 6:8499 doi: 10.1038/ncomms9499 (2015).

## Supplementary Material

Supplementary Information Supplementary Figures 1-20 and Supplementary Tables 1-2
