# Supplementary material for: STAT3-mediated IGF-2 secretion in the tumour microenvironment elicits innate resistance to anti-IGF-1R antibody
Source: Nat Commun. 2015 Oct 14;6:8499. doi: 10.1038/ncomms9499 (PMC4608384; doi:10.1038/ncomms9499)
Supplement: Supplementary Information — Supplementary Figures 1-20 and Supplementary Tables 1-2 [file ncomms9499-s1.pdf]

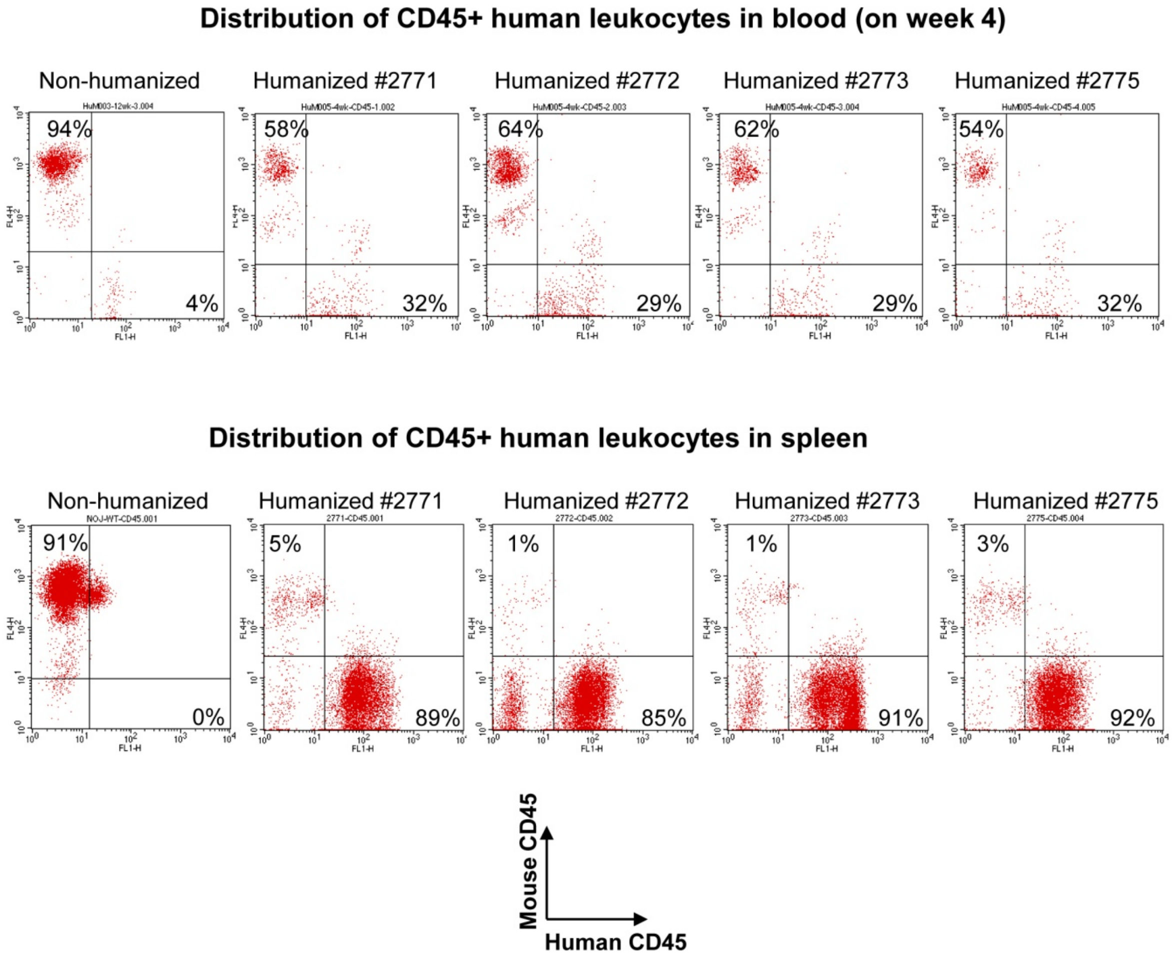

Supplementary Figure 1. Reconstitution of human-acquired lymphoid system in mouse NOD/SCID/Jak3^{null} mice were transplanted with human CD34^{+} hematopoietic stem cells. *(Top)* Four weeks after the transplantation peripheral blood was collected and the presence of human CD45^{+} leukocytes was analyzed by FACS. *(Bottom)* At the time of sacrifice, cells were isolated from the spleen of each mouse and the presence of human CD45^{+} leukocytes was analyzed by FACS.

a

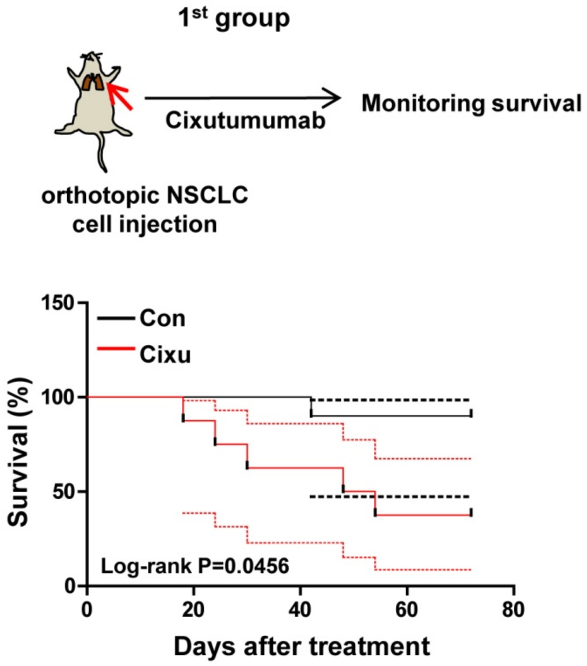
1st group
Cixutumumab
Monitoring survival
orthotopic NSCLC cell injection
Con
Cixu
Survival (%)
150
100
50
0
Log-rank P=0.0456
0
20
40
60
80
Days after treatment

b

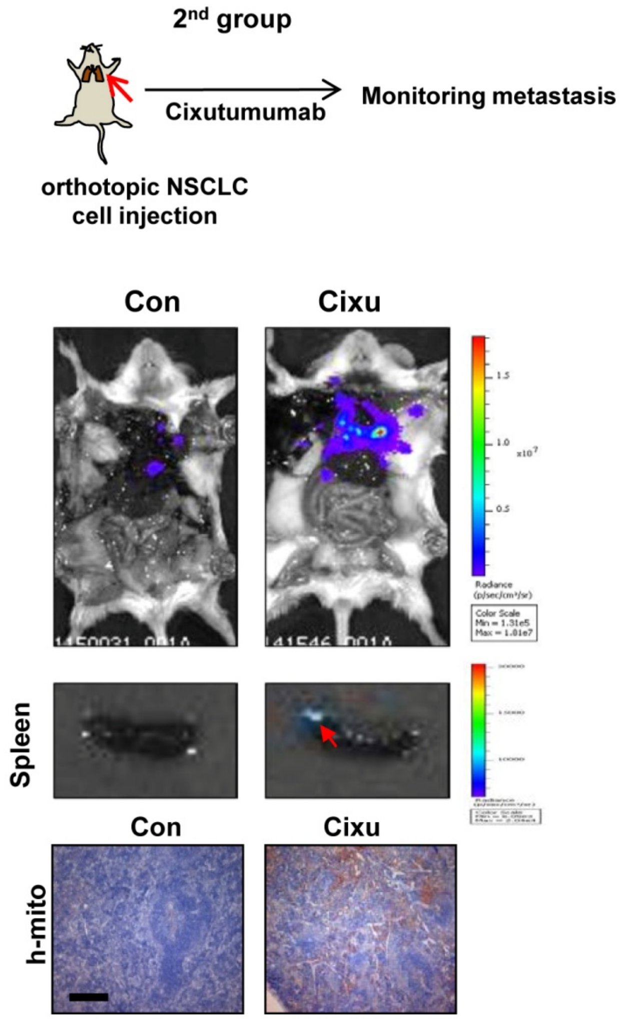
2nd group
Cixutumumab
Monitoring metastasis
orthotopic NSCLC cell injection
Con
Cixu
Radiance (p/sec/cm²/sr)
Color Scale
Min = 1.31e5
Max = 1.81e7
Spleen
Con
Cixu
h-mito

Supplementary Figure 2. Decreased survival and Increased metastasis after cixutumumab treatment in orthotopic lung tumor models (**a**) The mice bearing H1299-Luc tumors were treated with cixutumumab (10 mg kg^{-1}, intraperitoneally, once weekly) and monitored for survival analyses. Survival graph indicating decreased survival of mice with cixutumumab treatment. P=0.045 by two-sided Mantel-Cox log-rank test. Solid line indicates percent survival; dashed line indicates 95% confidence intervals. (**b, *Top***) Representative bioluminesecence images 28 days after the treatment visualizing the metastatic tumor burden in the spleen. (**b, *Bottom***) Representative images of excised spleen are presented to confirm metastatic tumors using anti-human mitochondria immunostaining (Scale bar: 200 μm). Cixu: cixutumumab; Con: control.

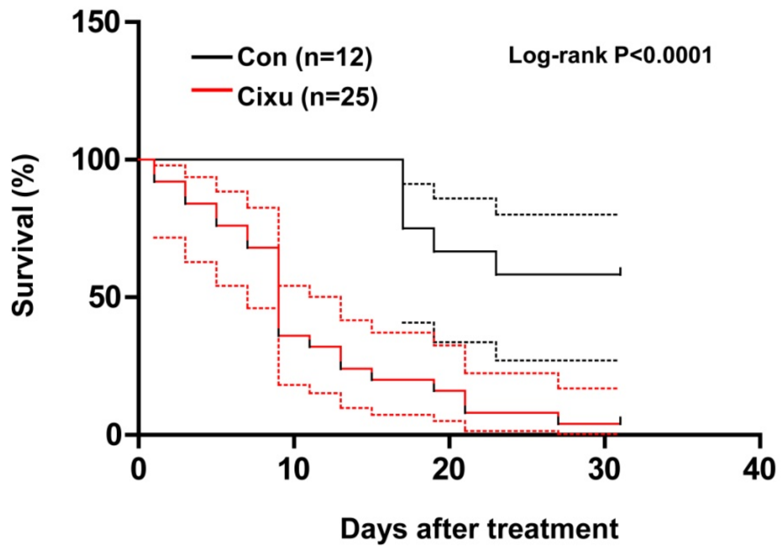

Supplementary Figure 3. Decreased survival after cixutumumab treatment in orthotopic head and neck tumor models 686LN-Luc cells were orthotopically injected into NOD/SCID mice, and the mice were treated with cixutumumab (10 mg kg^{-1}, intraperitoneally, once weekly). Survival graph indicating decreased survival of mice with cixutumumab treatment. $P < 0.0001$ by two-sided Mantel-Cox log-rank test. Solid line indicates percent survival; dashed line indicates 95% confidence intervals. Cixu: cixutumumab; Con: control.

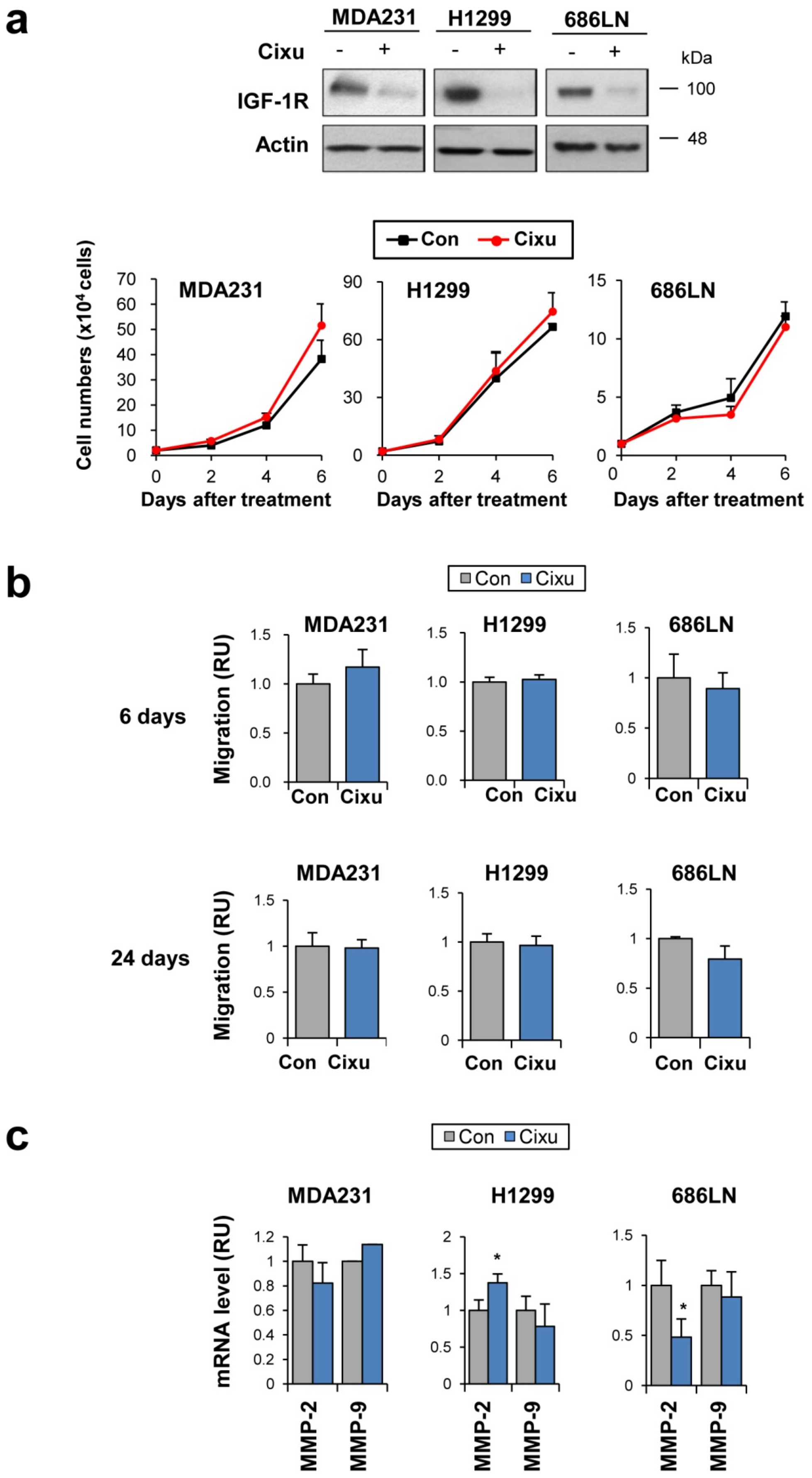
a
MDA231
H1299
686LN
Cixu
kDa
IGF-1R
Actin
100
48
Con
Cixu
Cell numbers (x10⁴ cells)
Days after treatment
b
6 days
24 days
Migration (RU)
c
mRNA level (RU)
MMP-2
MMP-9

Supplementary Figure 4. Effects of cixutumumab on proliferation, migration, and expression of metastasis-related genes in cancer cells (**a,** ***Top***) The indicated cancer cells were treated with cixutumumab for six days, and IGF-1R protein levels were determined by Western blotting. (**a,** ***Bottom***) Cell proliferation assay using cancer cells treated with cixutumumab. Values indicate the mean ±SD of four replicates of a single representative experiments. (**b**) Migration assay using cixutumumab-treated cancer cells. Cancer cells treated for six or 24 days were seeded in the upper well and incubated in conditioned media from NIH3T3 cells for 16-20 h. Each bar represents the mean relative unit (RU) ±SD of three identical wells of a single representative experiment. (**c**) Indicated cells were treated with cixutumumab for 6 days. MMP-2 and MMP-9 mRNA levels were analyzed by real-time PCR. Cixu: cixutumumab (25 μg ml^{-1}); Con: control.

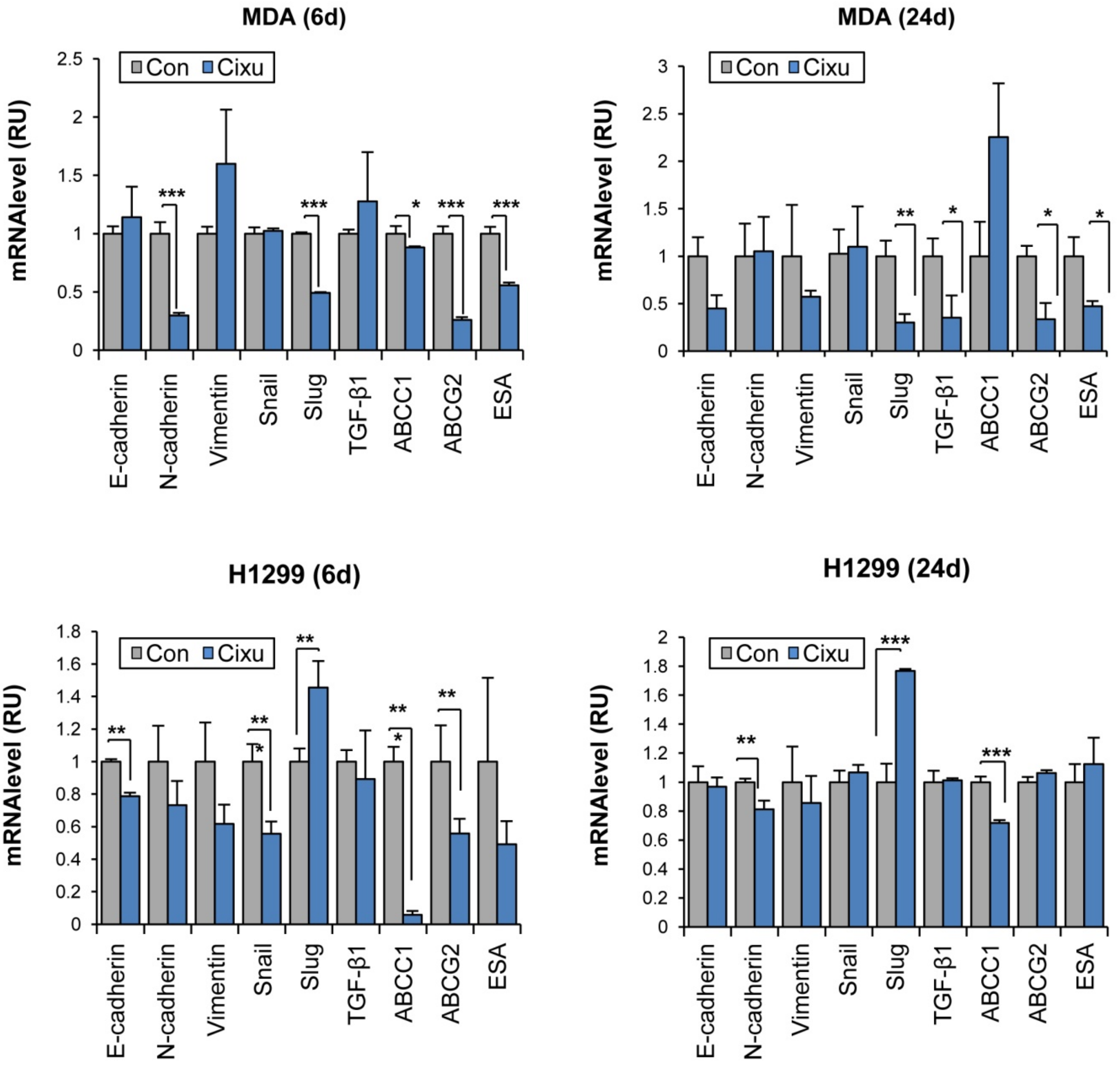

Supplementary Figure 5. Transcriptional changes of EMT and cancer stemness markers H1299 and MDA231 cells were treated with cixutumumab (25 μg ml^{-1}) for six or 24 days, and the expression of several EMT and cancer stem cell markers was determined by real-time PCR. Each bar represents the mean relative unit (RU) ±SD of three identical wells of a single representative experiment. *P < 0.05, **P < 0.01, and *** P <0.001 by two-sided Student's t-test. Cixu: cixutumumab; Con: control.

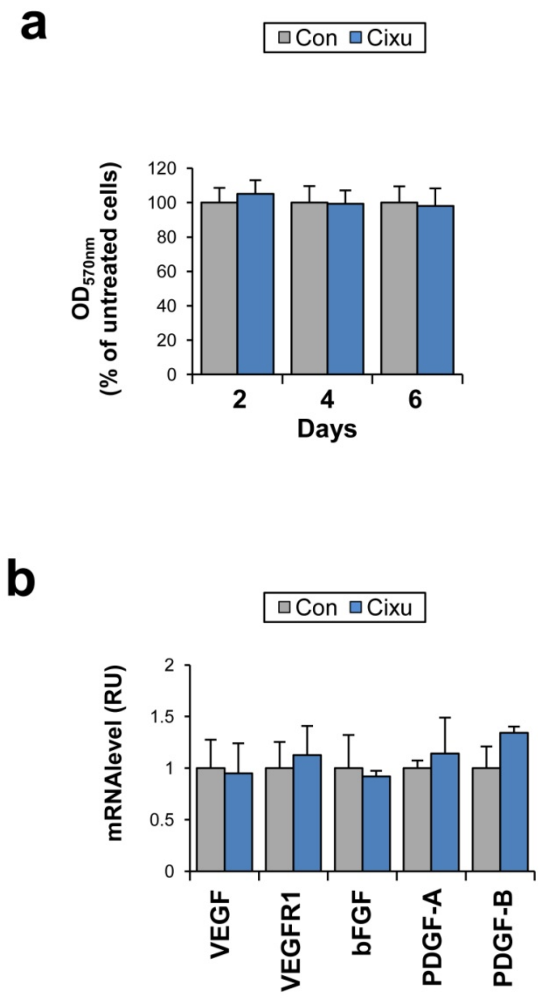

Supplementary Figure 6. Effects of cixutumumab on HUVECs (**a**) MTT assay for viability of HUVECs upon cixutumumab treatment. Each bar represents the mean ±SD of five identical wells of a single representative experiment. (**b**) Real-time PCR analysis of angiogenic factors in HUVECs treated with cixutumumab for six days. Bars indicate the mean relative unit ±SD of three replicates of a single representative experiment. Cixu: cixutumumab; Con: control.

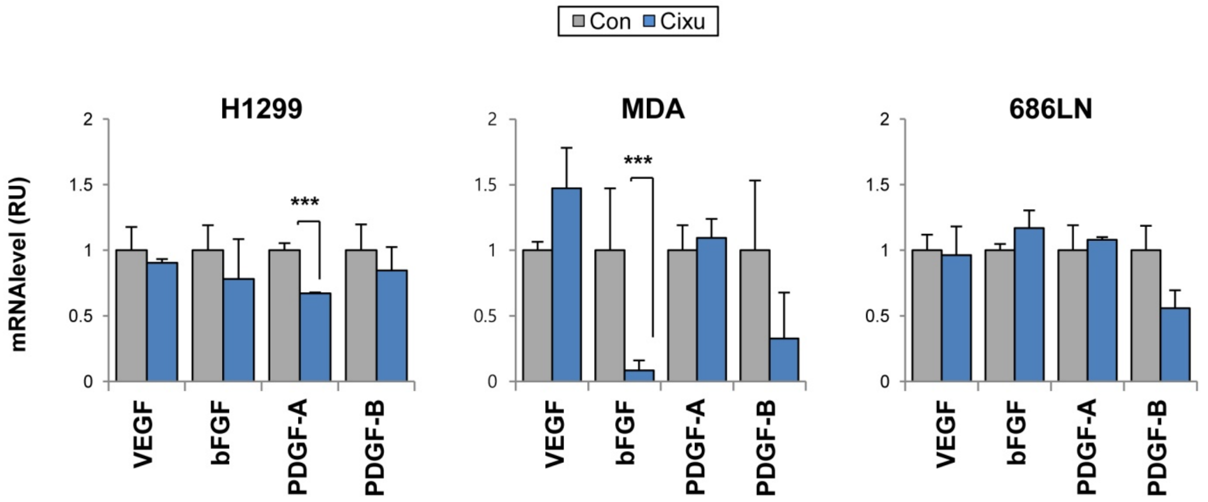

Supplementary Figure 7. Transcriptional changes of angiogenic factors in cixutumumab-treated cancer cells Real-time PCR analysis of angiogenic factors in indicated cancer cells treated with cixutumumab for six days. Bars indicate the mean relative unit (R) ±SD of three replicates of a single representative experiment. Cixu: cixutumumab; Con: control.

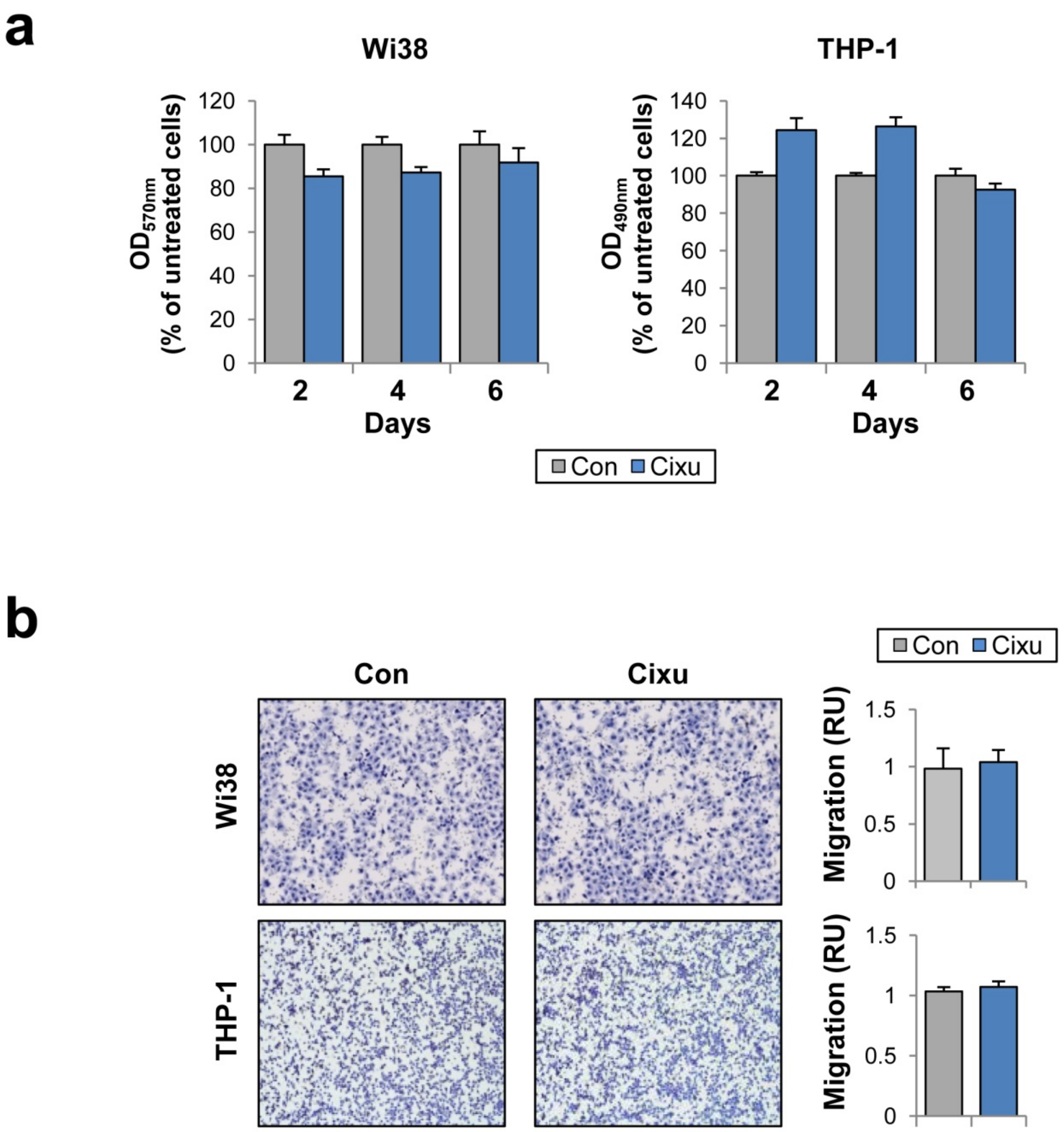

Supplementary Figure 8. Effects of cixutumumab on viability and migration in stromal cells (**a**) MTT (Wi38) and MTS (THP-1) assays using cixutumumab-treated cells. Each bar represents the mean ±SD of five identical wells of a single representative experiment. (**b**) Cells were treated with cixutumumab for six days, and cell migration was determined by using the transwell assay. *(Left)* Representative images of the migration assay for each cell type. *(Right)* Each bar represents the mean relative unit (RU) ±SD of three identical wells of a single representative experiment. Cixu: cixutumumab; Con: control.

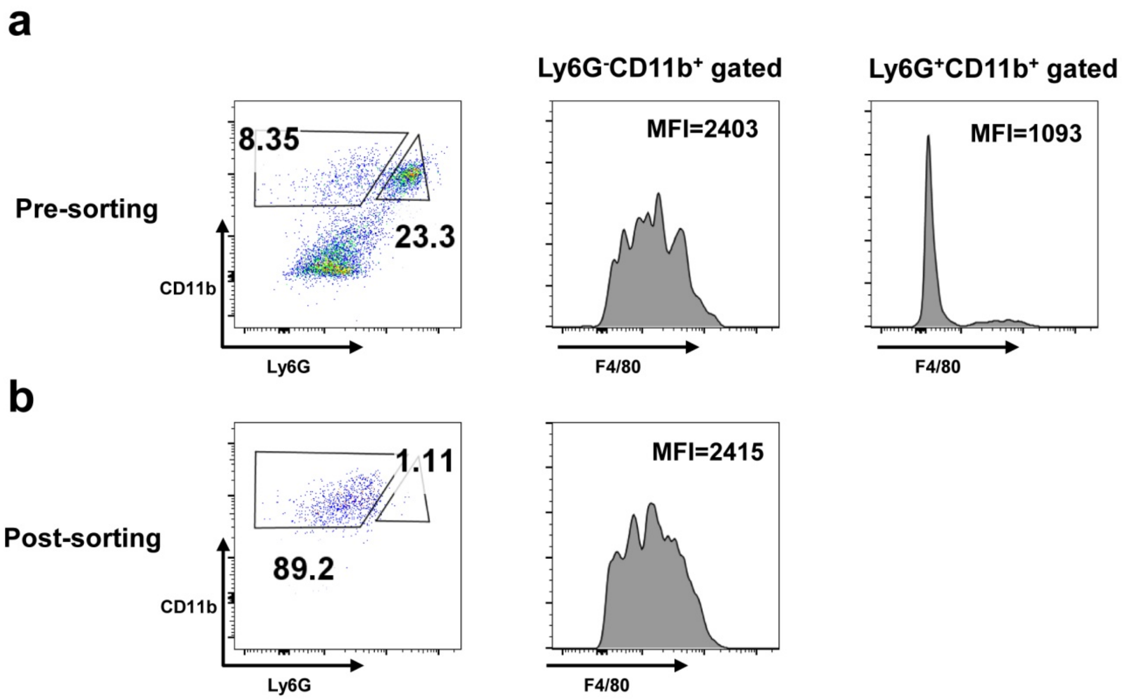

Supplementary Figure 9. Primary monocytes sorting and confirmation of the purity (**a**) CD11b positive population after the MACS separation using microbeads were further sorted using FACS Aria for granulocyte marker (Ly6G) and macrophage marker (F4/80). Ly6G^{-}/CD11b^{+} cells showed increased macrophage population compared to the Ly6G^{+}/CD11b^{+} cells. (**b**) After sorting the Ly6G^{-}/CD11b^{+} cells, the purity was analyzed at the same condition and confirmed high purity and consistent macrophage popuation. Numbers in the dot plot indicates percentage of the gated population. MFI: mean fluorescence intensity.

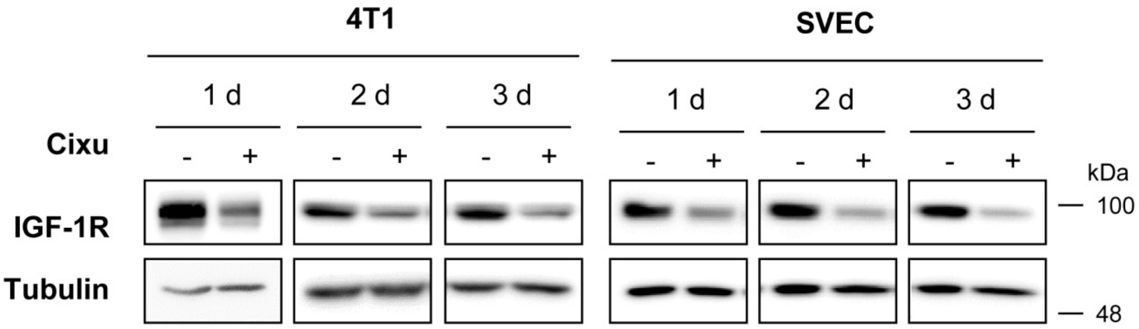

Supplementary Figure 10. Inhibition of murine IGF-1R expression by cixutumumab treatment The 4T1 and SVEC cells were treated with cixutumumab for one, two, and three days, and IGF-1R protein levels were determined by Western blotting.

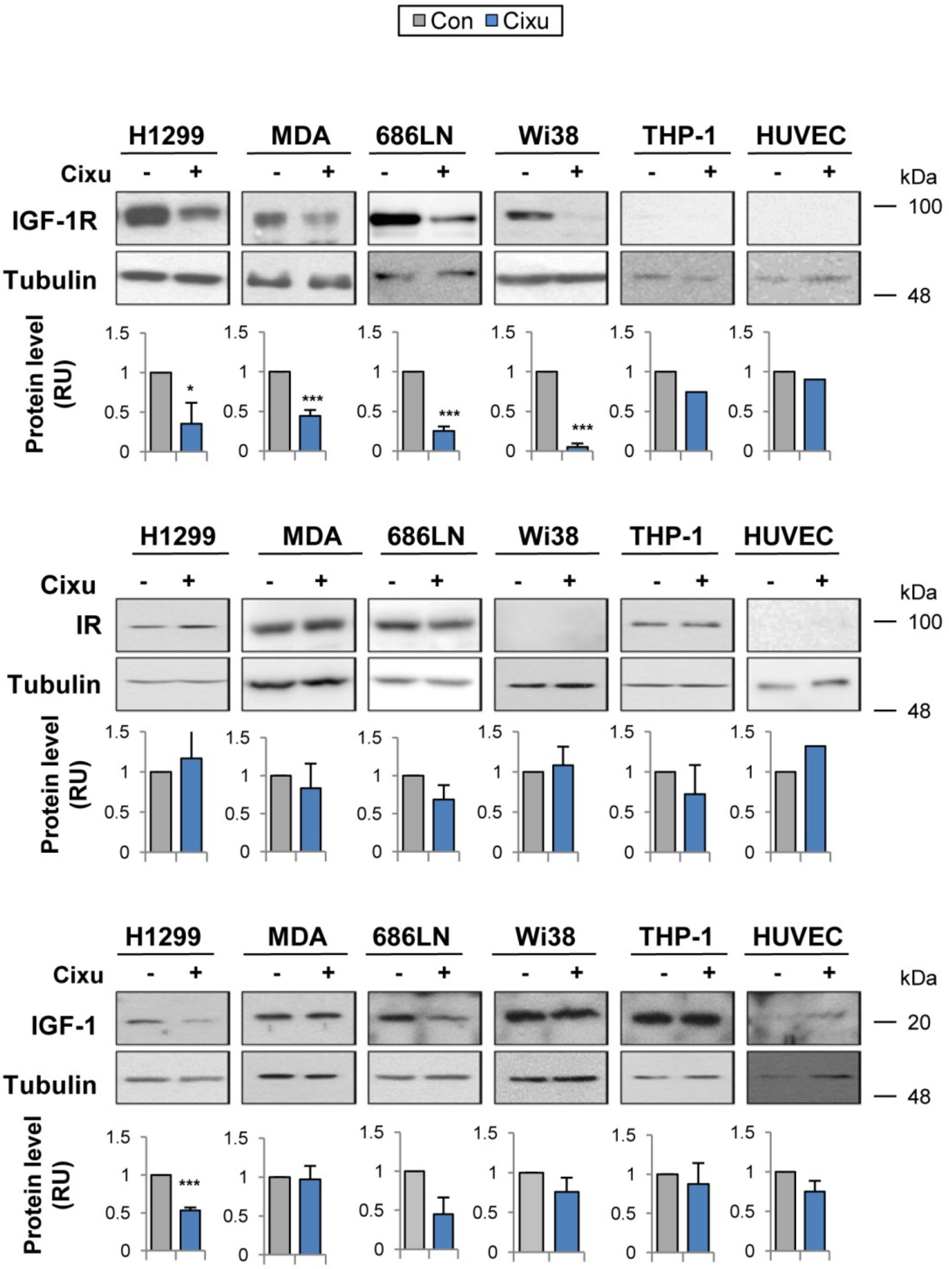

Supplementary Figure 11. Expression of ligand and receptors of IGF axis upon cixutumumab treatment Indicated cells were treated with cixutumumab for six days, and IGF-1R, IR, and IGF-1 protein levels were determined by Western blotting. Each graph below blots shows densitometric analysis quantifying the expression levels of the indicated proteins. Each bar represents the mean relative unit (RU) ± SD of the protein level of three independent experiments. **P* < 0.05, ***P* < 0.01, and ****P* < 0.001 by two-sided Student's t-test. Cixu: cixutumumab; Con: control.

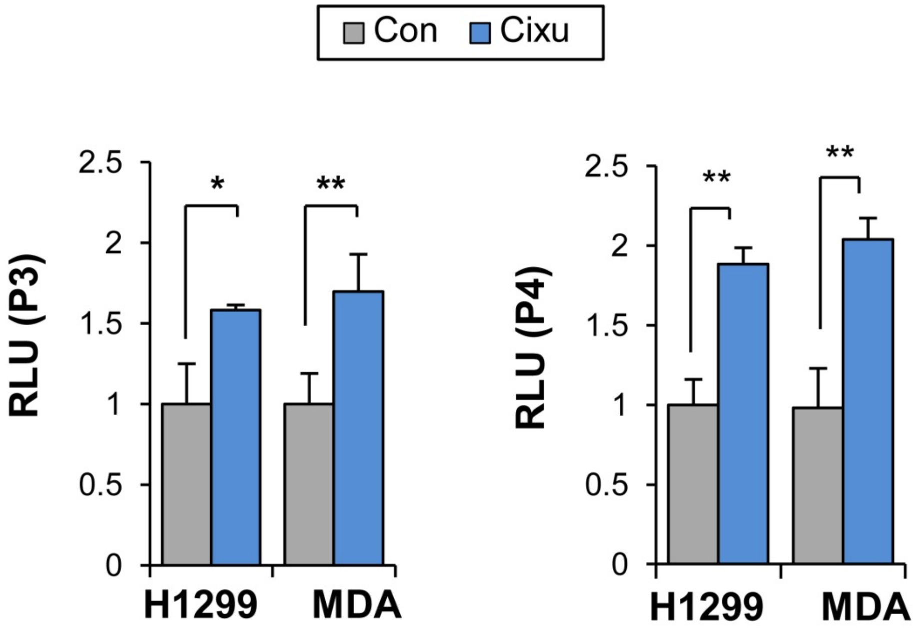

Supplementary Figure 12. IGF-2 promoter activities in cixutumuamb-treated cancer cells Indicated cells were treated with cixutumumab for six days, and each promoter activity was determined by luciferase assay. *(Left)* P3 promoter, *(Right)* P4 promoter. The values indicate the mean relative luciferase unit (RLU) ±SD. *P < 0.05 and **P < 0.01 by two-sided Student's t-test. Cixu: cixutumumab; Con: control.

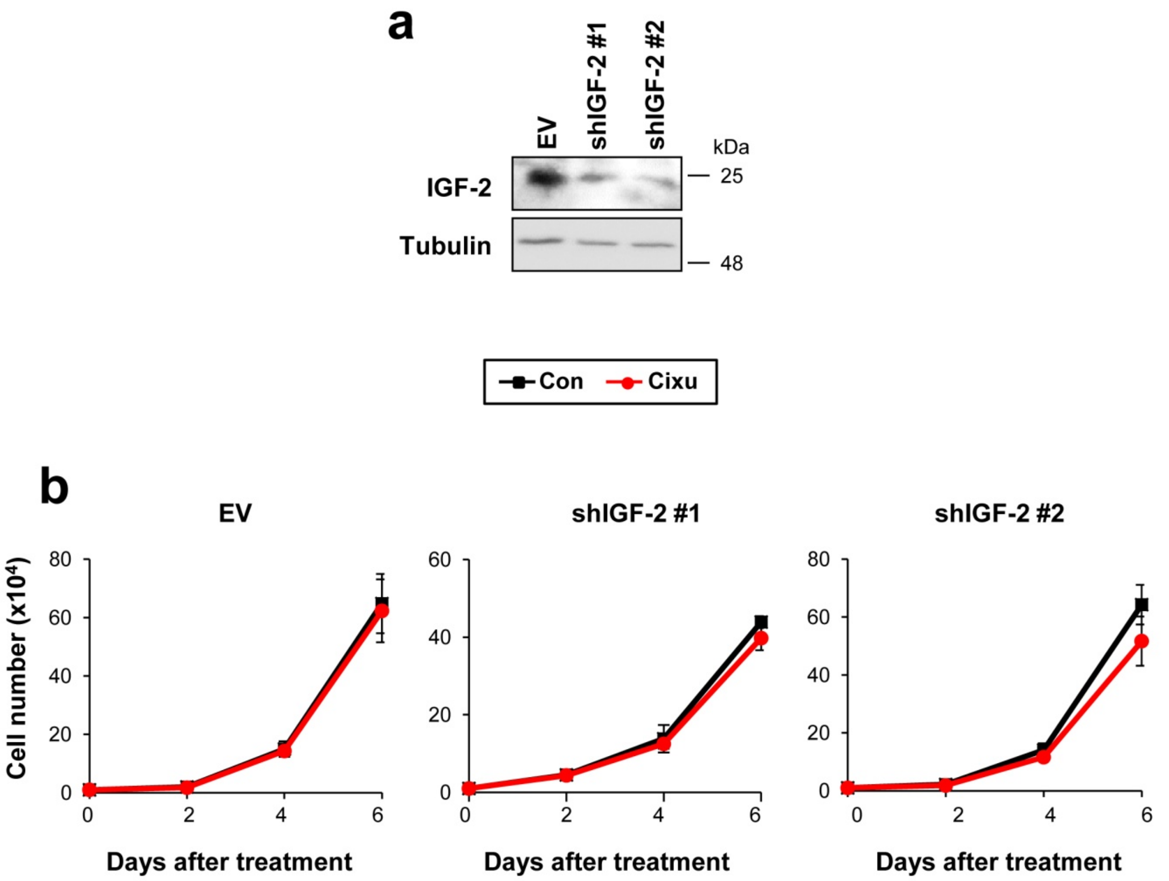

Supplementary Figure 13. Effects of cixutumumab on proliferation of IGF-2 knock downed H1299 cells (**a**) IGF-2 protein expression in empty vector or shIGF-2 transfected H1299 cells was analyzed by Western blotting. (**b**) Cell proliferation assay in empty vector (EV)- or shIGF-2-transfected H1299 cells treated with cixutumumab. Values indicate the mean ±SD of four replicates of a single representative experiments.

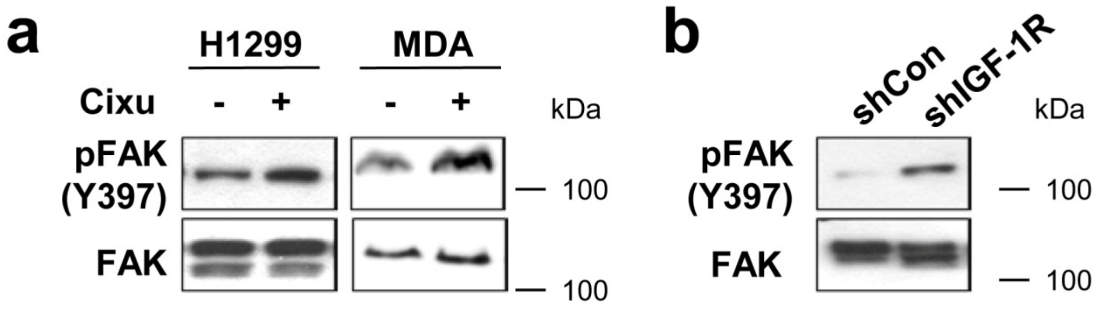

Supplementary Figure 14. Activation of FAK by cixutumumab (**a**) The indicated cancer cells were treated with cixutumumab (25 μg ml^{-1}) for 6 days, and the activation of FAK was determined by Western blotting. (**b**) Protein lysates from H1299 cells transfected with control vector (shCon) or shIGF-1R were analyzed for activation of FAK by Western blotting.

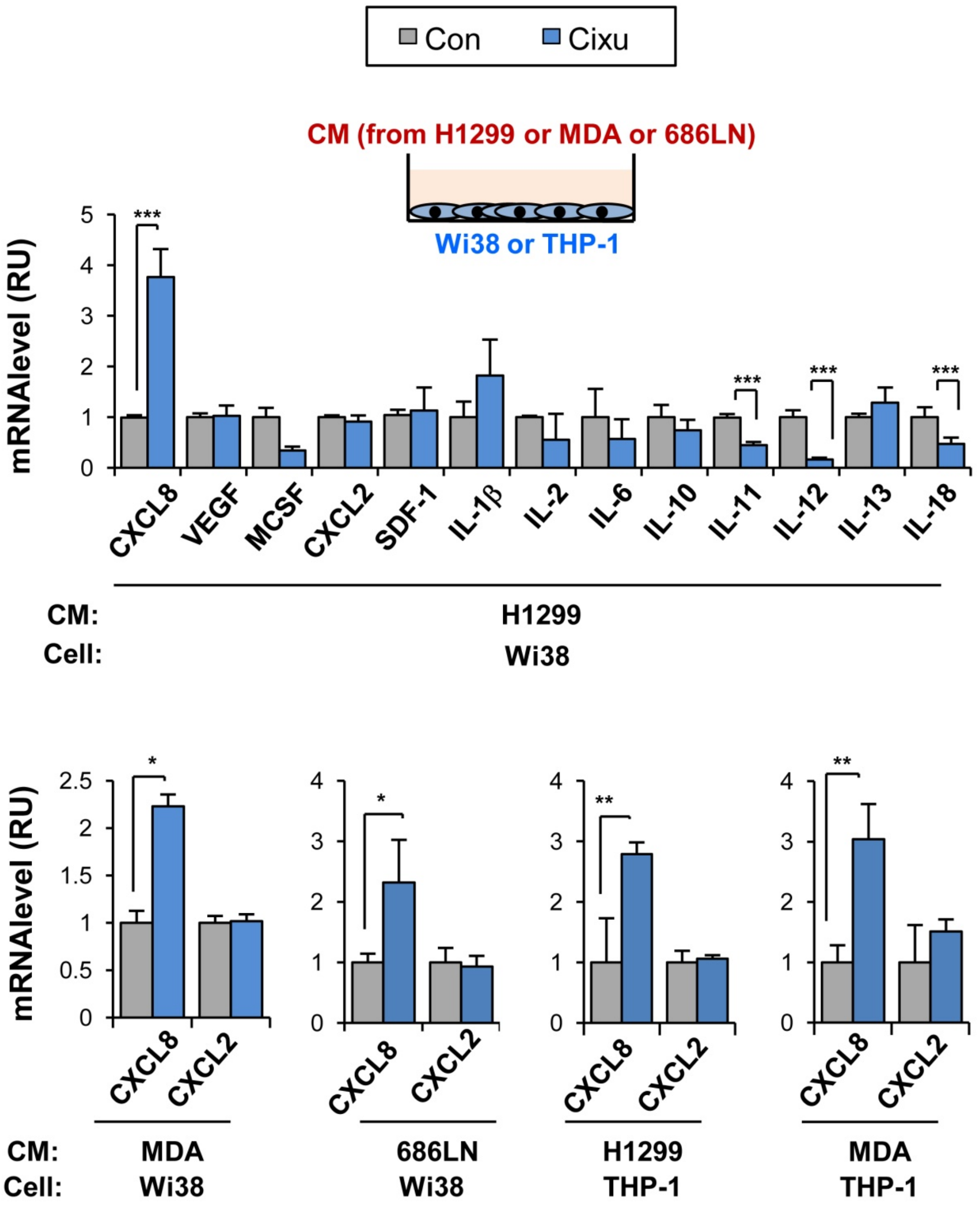

Supplementary Figure 15. Transcriptional changes of cytokines in stromal cells Wi38 or THP-1 cells were serum starved for 24 h, and then stimulated with CM from cixutumumab-treated cancer cells (final concentration to 1%). mRNA levels of several cytokines were analyzed by real-time PCR analysis. Each bar represents the mean relative unit ±SD of three identical wells of a single representative experiment. *$P < 0.05$, **$P < 0.01$, and ***$P < 0.001$ by two-sided Student's t-test. RU: relative unit; Cixu: cixutumumab; Con: control.

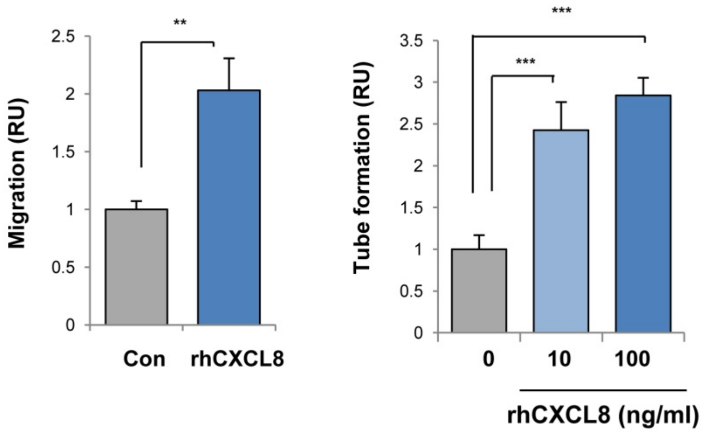

Supplementary Figure 16. Response of HUVECs to recombinant human CXCL8 *(Left)* Migration assay with rhCXCL8. HUVECs were seeded in basal media with or without rhCXCL8 (10 ng ml^{-1}) in the upper well and incubated for 6 h. *(Right)* HUVECs were seeded in basal media with or without rhCXCL8 onto a Matrigel-coated 96 well plate, and incubated for 10 h. Each bar represents the mean relative unit (RU) ±SD of three or four identical wells of a single representative experiment. ***P* < 0.01 and ****P* < 0.001 by two-sided Student's t-test.

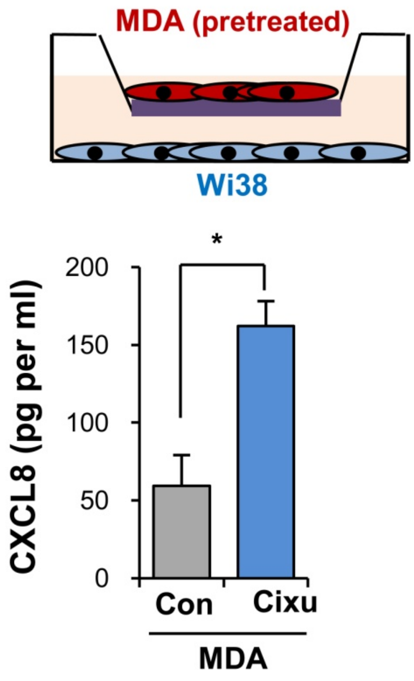

Supplementary Figure 17. Secretion of CXCL8 from cocultured Wi38 cells Wi38 cells were cocultured with cixutumumab-treated MDA231 cells, and CXCL8 secretion was determined by ELISA. Each bar represents the mean concentration ±SD of three replicates of a single representative experiment. Cixu: cixutumumab; Con: control. *P < 0.05 by two-sided Student's t-test.

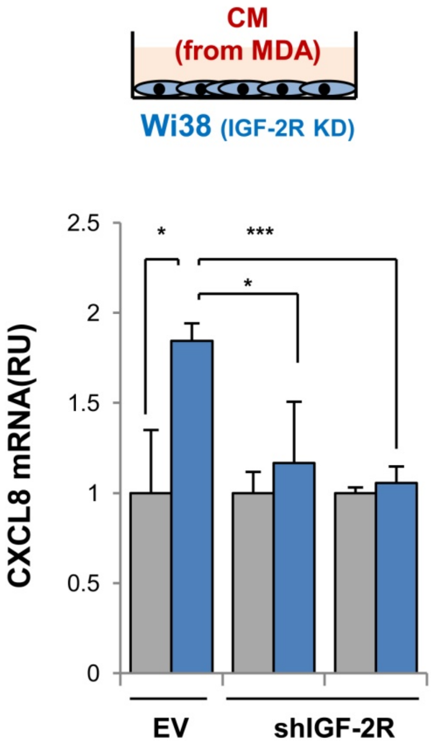

Supplementary Figure 18. IGF-1R-mediated CXCL8 transcription in CM treated Wi38 cells IGF-2R expression was reduced by shRNA in Wi38 cells and followed by treatment with CM from cixutumumab-treated MDA231 for 24 h. CXCL8 mRNA levels were determined by real-time PCR. Each bar represents the mean relative unit ±SD of three identical wells of a single representative experiment. * P <0.05 and *** P <0.001 by two-sided Student's t-test.

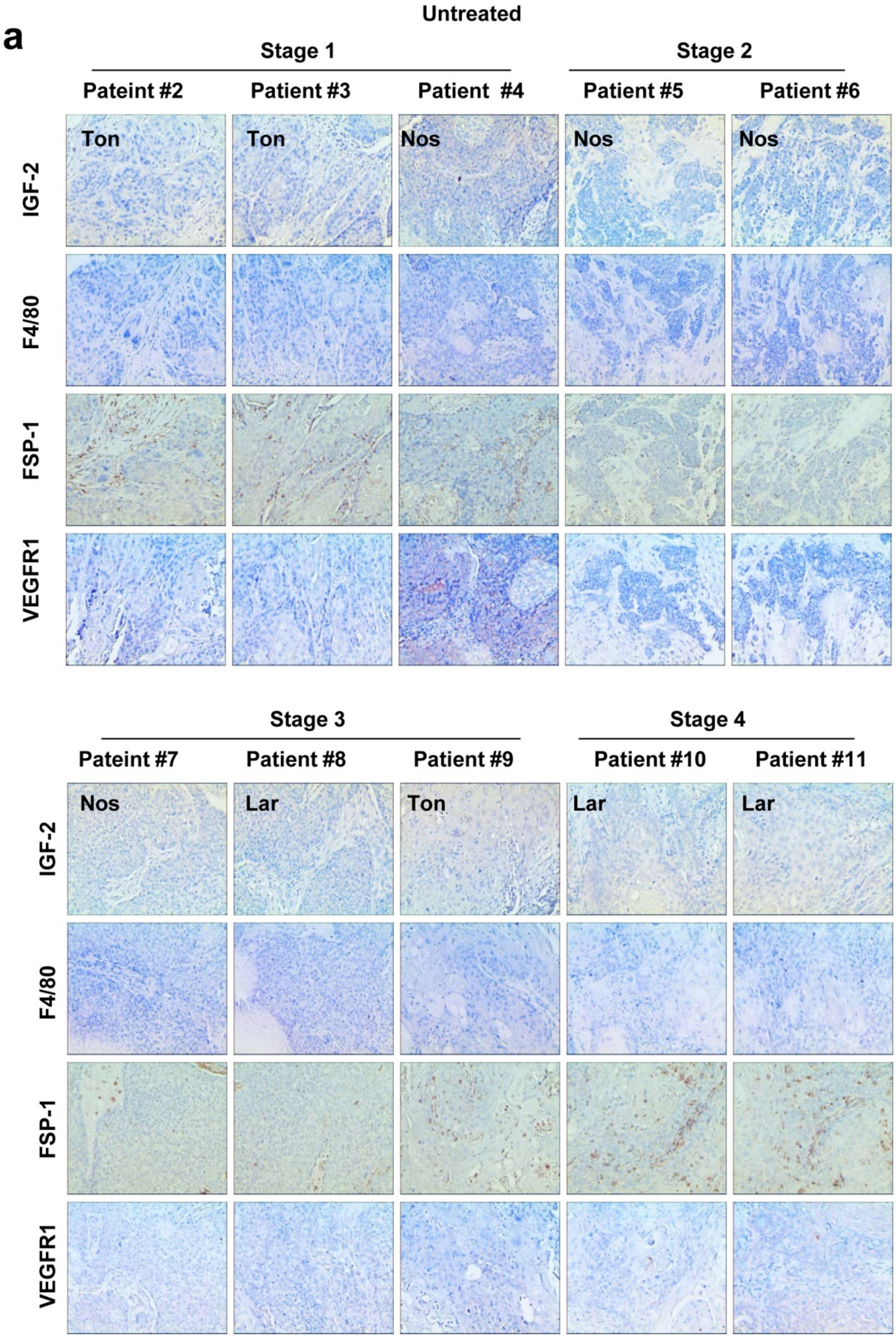

Supplementary Figure 19. All images of immunostaining for IGF-2, VEGR1, FSP-1, F4/80 in HNSCC patient tissue samples Inserted words indicate specific region of tumors. Lar: Larynx; Nos: Nose; Ton: Tongue.

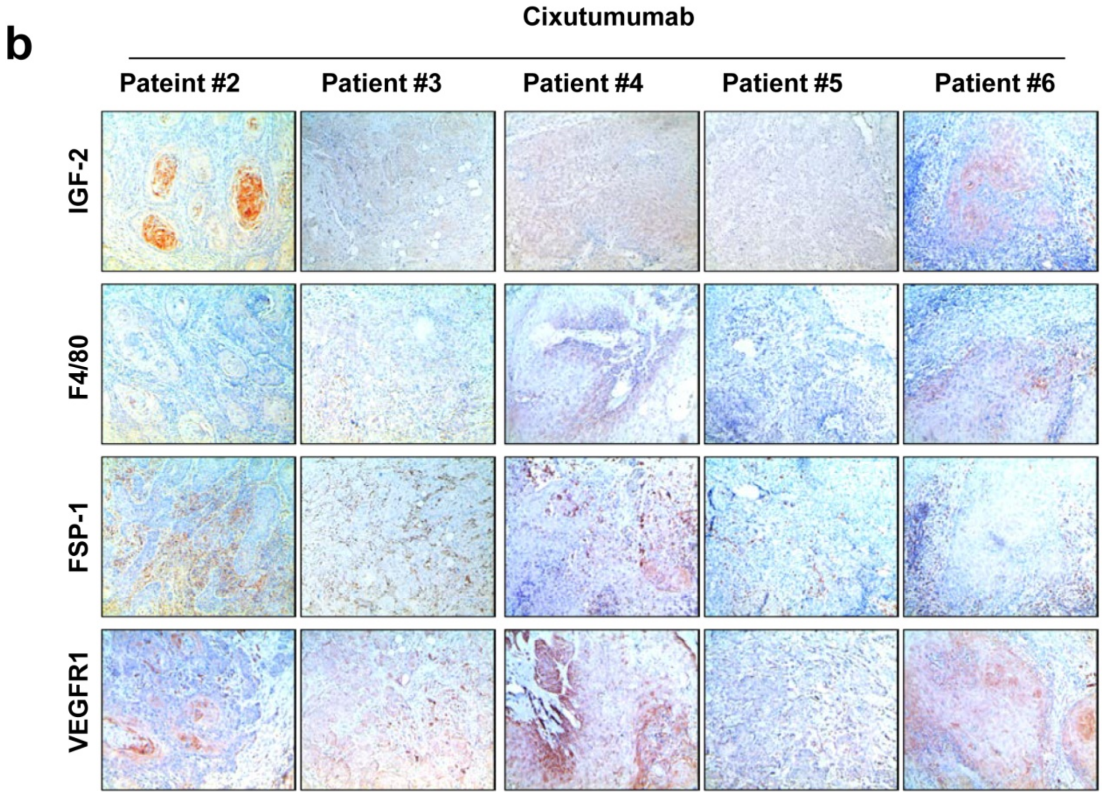

Supplementary Figure 19. Continued.

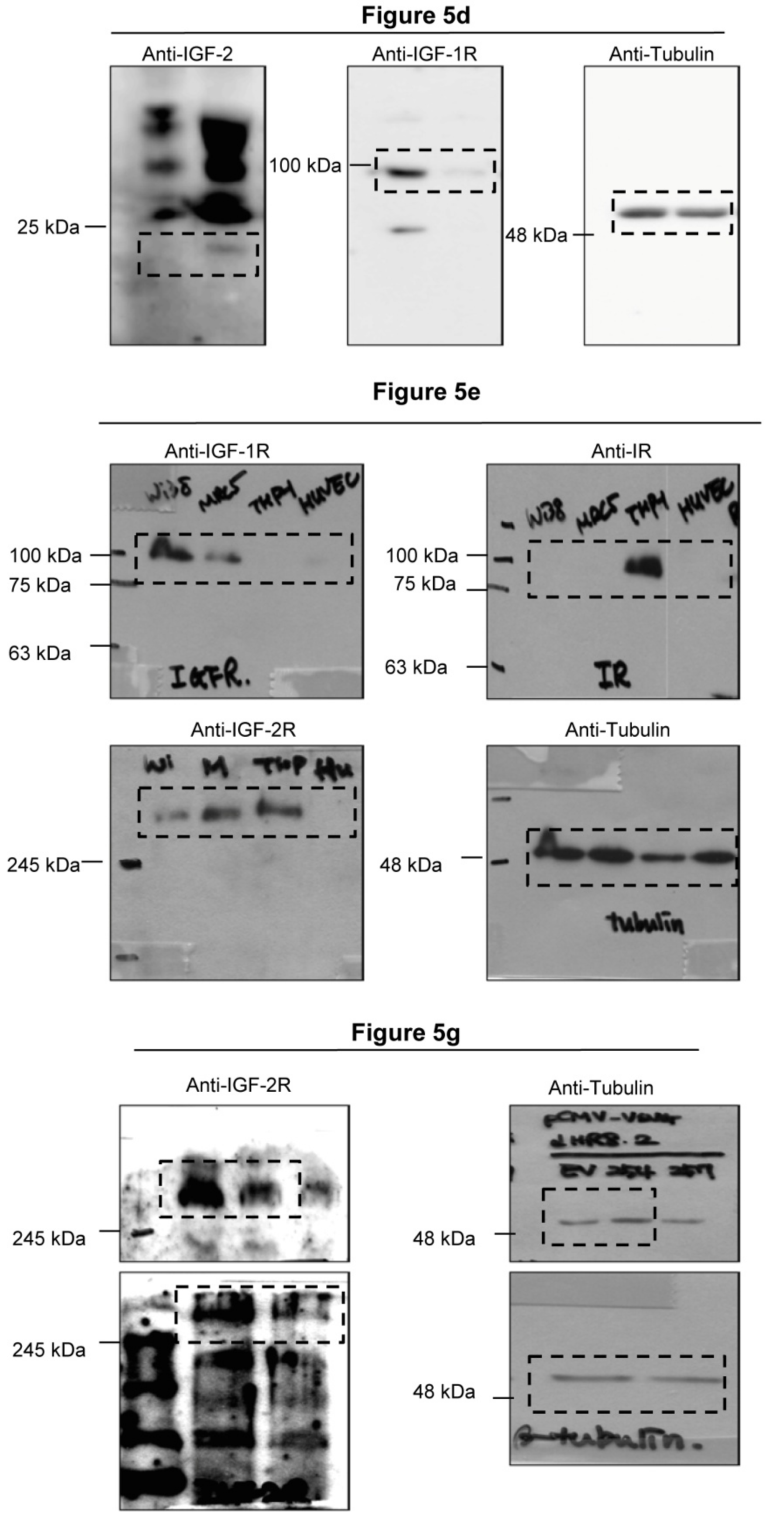

Supplementary Figure 20. Full blots of indicated figures

Figure 6b

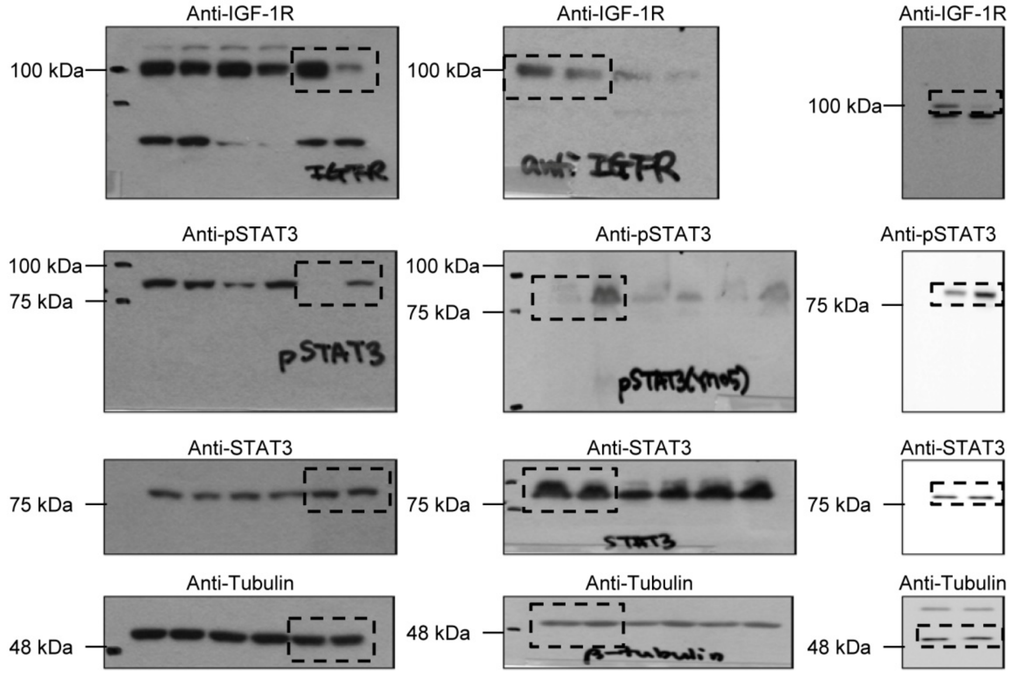

Figure 6c

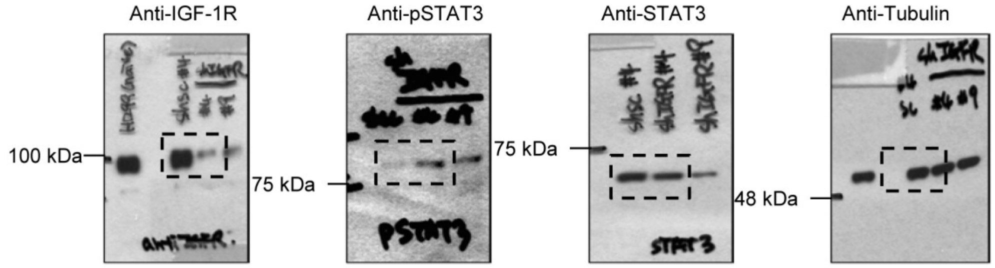

Figure 6d

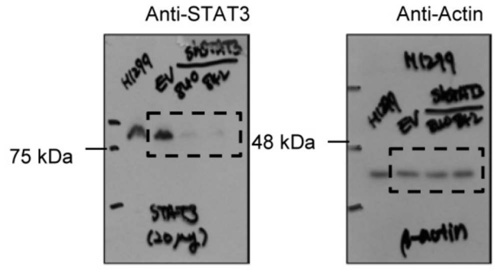

Figure 6e

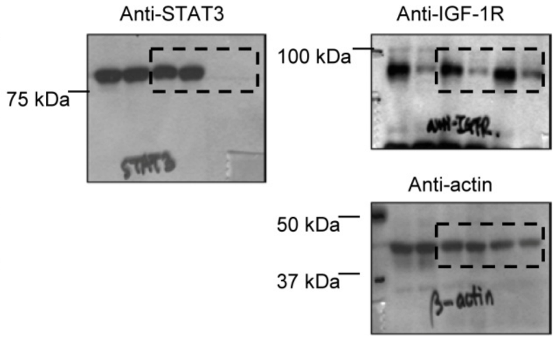

Supplementary Figure 20. Continued.

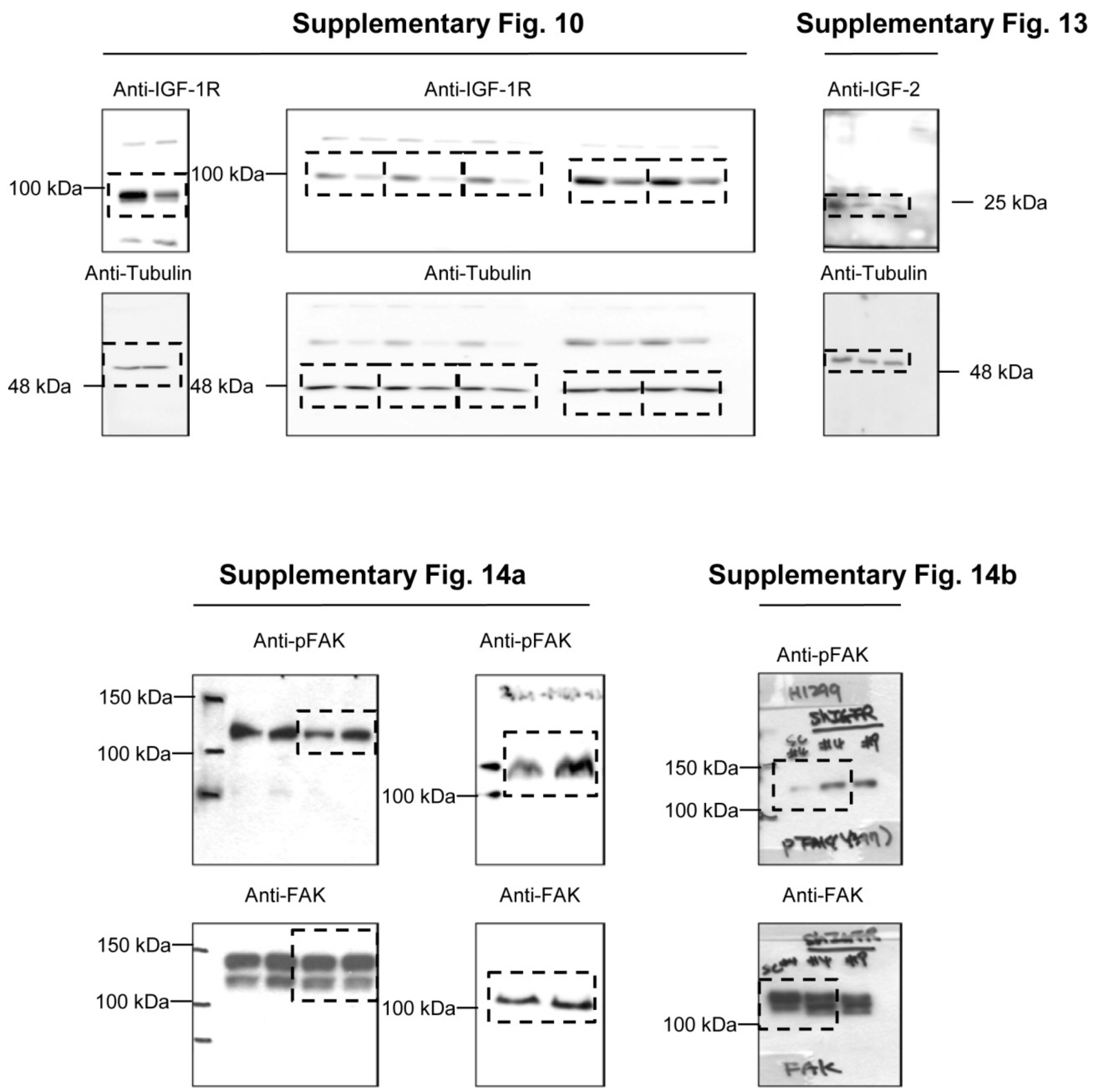

Supplementary Figure 20. Continued.

Supplementary Table 1. Primers for RT-PCR

target	sense	antisense
**Actin**	ACTACCTCATGAAGATC	GATCCACATCTGCTGGAA
**IGF-1**	TGCTCACCTTCACCAGCTCTGCCA	GTGTGGCGCTGGGCAGGGACAGA
**IGF-1R**	TGGGCCAAGAGTGAGATC	GTATTCAGCCTCCTCCTTC
**IGF-2**	TCGTGCTGCATTGCTGCTTACCG	GCTCACTTCCGATTGCTGGCCAT
**IGF-2R**	AGAAGCCTTAATTTGCACAG	TGCTTCTCAGCAATAGAACA
**IR**	AACCAGAGTGAGTATGAGGAT	CCGTTCCAGAGCGAAGTGCTT

Supplementary Table 2. Primers for real-time PCR

Target	sense	antisense
**E-cadherin**	GTCAGTTCAGACTCCAG CCC	AAATTCACTCTGCCCAGG ACG
**N-cadherin**	AGTCAACTGCAACCGT GTCT	AGCGTTCCTGTTCCACTCAT
**vimentin**	TCTACGAGGAGGAGATGC GG	GGTCAAGACGTGCCAGAGAC
**snail**	ACCACTATGCCGCGCTCTT	GGTCGTAGGGCTGCTGGAA
**slug**	AACAGAGCATTTGCAGACAGGTC	GCTACACAGCAGCCAGATTCC
**TGF-β**	GACACCAACTATTGCTTCAG	CAGGCTCCAAATGTAGGG
**ABCC1**	CTACCTCCTGTGGCTGAATC	ATCAGCTTGATCCGATTGTC
**ABCG2**	ACAGGTGGAGGCAAATCTTC	GCGGTGCTCCATTTATCAG
**ESA**	CAATGCAGGGTCTAAAAGCTG	CACCCATCTCCTTTATCTCAGC
**VEGF**	CCTGGTGGACATCTTCCAGGAGTACC	GAAGCTCATCTCTCCTATGTGCTGGC
**VEGFR1**	CACCACTCAAACGCT GACATGTA	GCTCGTTGGCGCACTCTT
**MMP-2**	ATAACCTGGATGCCGTCGT	AGGCACCCTTGAAGAAGTAG
**MMP-9**	GCTTTTCTTCTTCTCTGGGCGCC	CGGTCCTGGCAGAAATAGGCTTT
**bFGF**	TGTGCTAACCGTTACCTGGC	CGTTTCAGTGCCACATACCAA
**PDGF-A**	TTGGCCACCTTGACGCT	CCTGCCCATTCGGAGGAA
**PDGF-B**	TTTCTCACCTGGACAGGTCG	GAAGGAGCCTGGGTTCCCT
**IGF-1**	ATGTATTGCGCACCCCTCAA	GGGCACGGACAGAGCG
**IGF-2**	GCGGCTTCTACTTCAGCAG	CAGGTGTCATATTGGAACAAC
**IGF-1R**	CGATGTGTGAGAAGACCACCA	ACATTTTCTGGCAGCGGTTT
**IR**	AGGAGCCCAATGGTCTGA	GAGACGCAGAGATGCAGC
**CXCL2**	CGCCCAAACCGAAGTCATAG	AGACAAGCTTTCTGCCCATTCT
**CXCL8**	ACTGAGAGTGATTGAGAGTGGAC	AACCCTCTGCACCCAGTTTTC
**IL-10**	GAACCAAGACCCAGACATC	CATTCTTCACCTGCTCCAC
**IL-11**	GGACTGCTGCTGCTGCTGAAG	CACGGAAGGACTGTCTCTAAC
**IL-12**	TCGGCAGGTGGAGGTCAGC	CGCAGAATGTCAGGGGAAGTAGG
**IL-13**	AACATCACCCAGAACCAGAAG	CAGAATCCGCTCAGCATCC
**IL-18**	CCTCCTGGCTGCCAACTCT	GAAGCGATCTGGAAGGTCTGAG
**IL-1b**	TGATGGCTTATTACAGTGGCAATG	GTAGTGGTGGTGGGAGATTCG
**IL-2**	CAAGAATCCCAAACTCACCAG	CGTTGATATTGCTGATTAAGTCC
**IL-6**	GTGTTGCCTGCTGCCTTC	AGTGCCTCTTTGCTGCTTTC
**MCSF**	TTGGGAGTGGACACCTGCAGTCT	CCTTGGTGAAGCAGCTCTTCAGCC
**SDF-1**	CCGCGCTCTGCCTCAGCGACGGGAAG	CCTGTTTAAAGCTTTCTCCAGGTACT
**Actin**	GCGAGAAGATGACCCAGATC	GGATAGCACAGCCTGGATAG
